# Caspase activation in tumour‐infiltrating lymphocytes is associated with lymph node metastasis in oral squamous cell carcinoma

**DOI:** 10.1002/path.6145

**Published:** 2023-07-13

**Authors:** Priyanka G Bhosale, Robert A Kennedy, Fiona M Watt

**Affiliations:** ^1^ Centre for Gene Therapy & Regenerative Medicine King's College London London UK; ^2^ Faculty of Dentistry, Oral & Craniofacial Sciences King's College London London UK; ^3^ European Molecular Biology Laboratory Heidelberg Germany

**Keywords:** OSCC, CASPASE8, tumour‐infiltrating lymphocytes (TILs), immune evasion, apoptosis, cytotoxic T lymphocytes, lymph node metastasis, apoptotic T lymphocytes, T cell exhaustion

## Abstract

Oral squamous cell carcinomas (OSCCs) are genetically heterogeneous and exhibit diverse stromal and immune microenvironments. Acquired resistance to standard chemo‐, radio‐, and targeted therapies remains a major hurdle in planning effective treatment modalities for OSCC patients. Since Caspase 8 (CASP8) is frequently mutated in OSCCs, we were interested to explore a potential interaction between tumour‐infiltrating lymphocytes (TILs) and CASP8 activation using high‐content image analysis of human tumour (*n* = 32) sections. Despite the lymphocyte‐rich tumour microenvironment, we observed lower activation of CASP8 (0–10% of tumour area) and its downstream effector CASP3 (0–6%) in tumours than in normal oral epithelium. Conversely, we found apoptosis was high for all the lymphocyte subtypes examined (38–52% of lymphocytes within tumour islands). Tumours with higher Fas ligand (FasL) expression had a significantly higher proportion of cleaved CASP3/8 positive cytotoxic T cells within the tumour islands (*p* = 0.05), and this was associated with the presence of lymph node metastatic disease [odds ratio: 1.046, 95% confidence interval (1.002–1.091), *p* = 0.039]. Our finding of extensive activation of the extrinsic pathway of apoptosis in TILs, together with evidence of higher FasL in *CASP8* mutated tumours, may be useful in predicting the course of disease in individual patients. © 2023 The Authors. *The Journal of Pathology* published by John Wiley & Sons Ltd on behalf of The Pathological Society of Great Britain and Ireland.

## Introduction

Oral cancer is the 18th most common cancer worldwide [1], with oral squamous cell carcinoma (OSCC) accounting for more than 90% of cases. The net 5‐year survival for oral cancer falls in the region of 56.1% [2]. T stage (reflecting tumour size and depth of invasion) and N stage (reflecting nodal metastases), together with histopathological features, including perineural invasion and margin status, are established prognostic factors [3]. The impact of the tumour immune response has become of increased interest and importance with the introduction of immunomodulatory therapies for the treatment of OSCC, such as Pembrolizumab, that target programmed cell death protein 1 (PD‐1) located on lymphocytes, which is a receptor for programmed death ligand 1 (PD‐L1) [4].

Studies examining tumour‐infiltrating lymphocytes (TILs) in OSCC using routine stains have found that a lower density is associated with local recurrence [5] and reduced survival [5, 6, 7]. Different lymphocyte subtypes, including cytotoxic T cells (Tc, CD8 positive), T helper cells (Th, CD4 positive), regulatory T cells (Treg, FOXP3 positive), natural killer cells (NK, CD56 and CD57 positive), and B cells (CD79a or CD20 positive), have all received attention for their importance in the prognosis of a wide range of cancers. However, studies examining lymphocyte subtypes in OSCC are limited and have produced mixed results [8, 9, 10, 11].

Detailed immune profiling of OSCC could lead to the development of prognostic and predictive biomarkers that could improve targeted therapies and enhance patient survival. Studies of the molecular processes and cellular outcome of interactions between TILs and OSCC are, thus, necessary. The extrinsic pathway of apoptosis is of particular interest in this regard. Fas ligand (FasL) is expressed by lymphocytes and on binding to its receptor on target cells leads to activation (cleavage) of Caspase‐8 (cCASP8) and the downstream effector Caspase‐3 (cCASP3), resulting in the induction of apoptosis [12]. The existence of frequent inactivating *CASP8* mutations in OSCC [13, 14, 15, 16, 17] would suggest that a subset of OSCC is protected from TIL‐mediated killing.

In this study we examined the density of subtyped lymphocytes together with activation of CASP3 and CASP8 in OSCC and TILs using automated digital analysis of multiplex immunofluorescence performed on whole‐slide human tumour sections. Patterns of FasL expression were examined by immunohistochemistry (IHC). In addition, the association of CASP3 and CASP8 activation with keratinisation or terminal differentiation was explored. OSCC cell lines that have been subjected to next‐generation sequencing were tested to examine the impact of *CASP8* mutations on activation of CASP3 and CASP8 by FasL.

## Materials and methods

### Patient cohort

OSCC samples (*n* = 32) and normal oral mucosa controls (*n* = 5) were used with Health Research Authority (HRA) approval and ethical review (REC reference 17/LO/18 24) and were retrieved from the Guy's Hospital (Great Maze Pond, London, UK) archive together with pathological data, treatment data, and outcome data. Oral dysplasia samples (*n* = 13), retrieved from Barts Health NHS Trust, London, UK, were used with HRA approval and ethical review (REC reference 18/WM/0326). The histopathological evaluation (including differentiation status, grade, and PNI) was performed by two independent pathologists (original reporting pathologist and RAK).

### Cell cultures

Five OSCC‐derived lines (SJGs) and normal oral mucosa‐derived lines [oral keratinocytes (OKs)] were cultured on a feeder layer of J2 3T3 cells in complete FAD medium (Thermo Fisher Scientific, Waltham, MA, USA), as described previously [15].

### IHC and multiplex immunofluorescence staining

Paraffin‐embedded formalin‐fixed tissue sections were heated at 56 °C for 1 h, followed by deparaffinisation and rehydration using a series of washes in xylene (Thermo Fisher Scientific) (×2), 100% ethanol (Sigma‐Aldrich, St. Louis, MO, USA) (×2), 70% ethanol (×2), and PBS (Sigma‐Aldrich). The sections were subjected to antigen retrieval for 16 min in either a citrate‐based (pH 6.0) or Tris‐based (pH 9.0) antigen unmasking solution (Vector Laboratories, Newark, CA, USA), as detailed in supplementary material, Table S1. Sections were blocked for 1 h at room temperature with blocking solution containing 10% foetal bovine serum (Sigma‐Aldrich), 2% BSA (Sigma‐Aldrich), 0.02% fish skin gelatin (Sigma‐Aldrich), 0.05% Triton ×100 (Sigma‐Aldrich), and 0.05% Tween 20 (Sigma‐Aldrich) in PBS. Sections were then incubated overnight at 4 °C with commercially available and validated primary antibodies (supplementary material, Table S1). For multiplex staining, sections were incubated with the following primary antibody cocktails: CD4+cCASP3+Keratin14(K14); CD8+cCASP3+K14; CD57+cCASP3+K14; CD79a+cCASP3+K14; cCASP3+K14+CD45+CD79a; cCASP8+K14+CD45+CD79a; or PD‐L1+K14+CD45+CD79a. The following day, the sections were incubated for 1 h at room temperature in a cocktail of secondary antibodies (AlexaFluor 488, 555, and 647) (supplementary material, Table S1). DAPI (Life Technologies, Carlsbad, CA, USA) was used as a nuclear counterstain. Slides were mounted using ProLong Gold antifade reagent (Life Technologies).

For IHC, a 3,3′‐diaminobenzidine (DAB)‐based ImmPRESS Polymer Detection Kit (Vector Laboratories) was used. Staining was performed according to the manufacturer's instructions [ImmPRESS® HRP Horse Anti‐Rabbit IgG Polymer Detection Kit (MP‐7401); ImmPACT® DAB Substrate, Peroxidase (HRP) (SK‐4105); BLOXALL® Endogenous Blocking Solution (SP‐6000‐100); Vector Laboratories]. Nuclei were counterstained with haematoxylin (Sigma‐Aldrich). Slides were mounted using VectaMount® AQ Aqueous Mounting Medium (Vector Laboratories).

Several positive and negative controls for antibody staining were included. The antibody to keratin 14 was validated as staining the basal layer of healthy epidermis. The lymphocyte‐specific antibodies were clinical grade. Sections of tonsil or lymph node served as positive controls for cCASP3 and cCASP8 staining and for markers of specific lymphocyte subtypes, given their localisation to distinct regions of those tissues (supplementary material, Figure S1A,B). Oral lichen planus tissue served as a positive control for apoptotic epithelial cells (not shown). For each antibody, unlabelled regions of sections served as a negative control.

### Multiplex imaging (high content imaging and digital pathology)

Immunofluorescence images were acquired using an Operetta® CLS™ high content analysis system (PerkinElmer, Waltham, MA, USA) with a 20× water immersion objective (NA 1.0). An automated high‐content analysis (HCA) pipeline was built in‐house using Harmony™ 4.8 software (PerkinElmer). We used a flatfield correction algorithm to generate uniform intensity profiles based on positive intensity values. Supplementary material, Figures S2–S4 provide details of the pipeline used for image quantification. Keratin 14, which is extensively expressed in tumour cells (except for the most highly differentiated/keratinised areas), was used to identify tumour regions for the HCA pipeline. An antibody cocktail containing anti‐CD45 + CD79a was used to label the total immune population for subsequent masking of immune subpopulations expressing cCASP3, cCASP8, and PD‐L1 in the HCA pipeline (supplementary material, Figures S2–S4).

IHC and H&E‐stained slides were scanned using a NanoZoomer 2.0RS Digital Slide Scanner (Hamamatsu Photonics K.K., Shizuoka, Japan) at 0.23 μm/pixel, 40× high‐resolution (Brightfield) mode. FasL immunohistochemical staining was assessed by the study pathologist. The FasL staining intensity (representative images in supplementary material, Figure S1C for 0, no staining; 1, weak staining; 2, moderate staining; 3, strong staining) and percentage tumour area stained were graded, and H‐scores were calculated using the following formula: H‐score = [0 × (% tumour area with score 0) + 1 × (% tumour area with score 1) + 2 × (% tumour area with score 2) + 3 × (% tumour area with score 3)] [18].

### Caspase activity assay

OSCC‐derived cells (SJGs) and OK cells were seeded on Corning® Rat Tail Collagen I (Corning, NY, USA) coated 96‐well plates (30,000 cells/well). The next day, cells were treated for 6 h with 0–400 ng/ml human FasL (Sigma‐Aldrich). Measurements of caspase activity were performed using the Caspase‐Glo® 3/7 Assay System (Promega, Madison, WI, USA) and the Caspase‐Glo® 8 Assay System (Promega), following the manufacturer's instructions.

### Statistical analysis

Tests used in statistical analyses are indicated in the figure legends; *p* < 0.05 was considered significant. Low and high expression or categorisation for each parameter analysed was performed based on median values. Analyses were carried out using IBM® SPSS® Statistics software (version 27) (IBM, Armonk, New York, USA), and GraphPad Prism software (version 8.4.3; Graphpad Inc., San Diego, CA, USA). The *p* values in the figures are represented as *p < 0.05, **p < 0.01, ***p < 0.001, ****p < 0.0001, ns: not significant.

## Results

### Reduced activation of CASP3 and CASP8 in OSCC tumour cells

**Figure 1:**
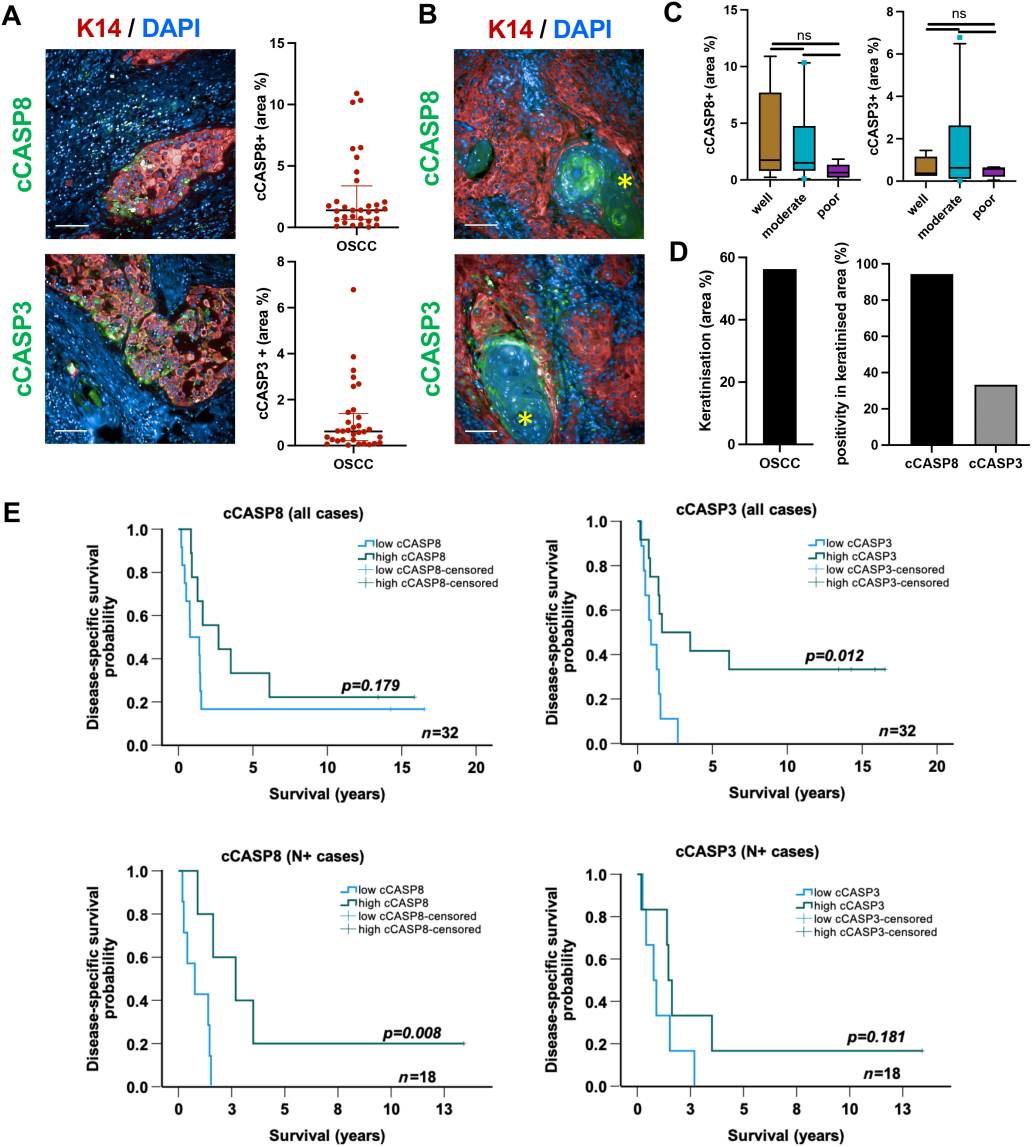
Activation of CASP8 and CASP3 expression in a small subset of human OSCC. (A) cCASP8 or cCASP3 (green) and keratin 14 (red) expression in OSCC. (B) cCASP8 (green) expression (marked with asterisk) in keratinised, well‐differentiated areas. (C and D) Box plots showing CASP3 and CASP8 activation based on tumour differentiation status (well, moderate, and poor differentiation) and in keratinised areas. (E) Kaplan–Meier plots of DSS (log‐rank test) in patients with high or low cCASP8 or cCASP3 expression in total study cohort (*n* = 32) or cases with lymph node metastasis (N+, *n* = 18). Data shown as mean ± SD; one‐way ANOVA with Šidák's multiple comparisons test. ns, not significant. Scale bars, 100 μm; *n* = 32.

The demographic details of the patient samples used in this study are provided in Table 1. Sequential cases of OSCC measuring more than 40 mm with at least 2 years’ follow‐up data were selected from the Guy's Hospital oral pathology archive. Primary OSCC tumours (*n* = 32) with varying degrees of lymphocyte infiltrate as evaluated by a pathologist (supplementary material, Figure S5A) were assessed for apoptotic tumour cells. Multiplex immunofluorescence was performed using cleaved CASP3 (cCASP3) and cleaved CASP8 (cCASP8) markers to evaluate apoptosis. HCA showed low activation of CASP3 and CASP8, ranging between 0–6% and 0–10% of the tumour (keratin 14‐positive) area respectively (Figure 1A and supplementary material, Figure S5B). Eighteen cases showed keratinisation; 17 (94%) of these showed activation of cCASP8 in the keratinised areas (Figure 1B–D). cCASP3 was detected in the keratinised areas in six (33%) of the 17 cases (supplementary material, Table S2). High levels of cCASP3 were associated with better disease‐specific survival (DSS) in the total patient cohort [hazard ratio (HR): 3.495; 95% confidence interval (CI): 1.189–10.273; *p* = 0.012] (Figure 1E). High levels of cCASP8 were associated with better survival among the cases with lymph node metastases (HR: 9.276; 95% CI: 1.089–79.04; *p* = 0.008) (Figure 1E).

**Table 1:** Demographics of study cohort

Characteristic	Patients with OSCC, *n* = 32 (%)
Site of origin	
**Oral cavity (comprising all sites)**	**32 (100%)**
Tongue	12 (37.5)
Buccal mucosa	5 (15.6)
Floor of mouth	14 (43.8)
Alveolar ridges	1 (3.1)
Age at diagnosis (year)	
Median (IQR)	59.3 (50.9–66.3)
Gender	
Male	23 (71.9)
Female	9 (28.1)
Stage	
Stage I and II	0 (0)
Stage III and IV	32 (100)
T classification	
T3	15 (46.9)
T4	17 (53.1)
N classification	
Lymph node metastasis negative (N0)	14 (43.8)
Lymph node metastasis positive (N+)	18 (56.3)
Tumour differentiation	
Well	5 (15.6)
Moderate	22 (68.8)
Poor	5 (15.6)
Recurrence	
Yes	16 (50)
No	16 (50)
Survival status	
Alive	5 (15.6)
Disease‐specific death	17 (53.1)
Non‐disease‐specific death	10 (31.3)

Bold value represents the total number of study samples while those listed below are the number of samples in each category.

IQR, interquartile range; OSCC, oral squamous cell carcinoma.

### TIL density and PD‐L1 expression are not correlated with activation of CASP8 in OSCC

**Figure 2:**
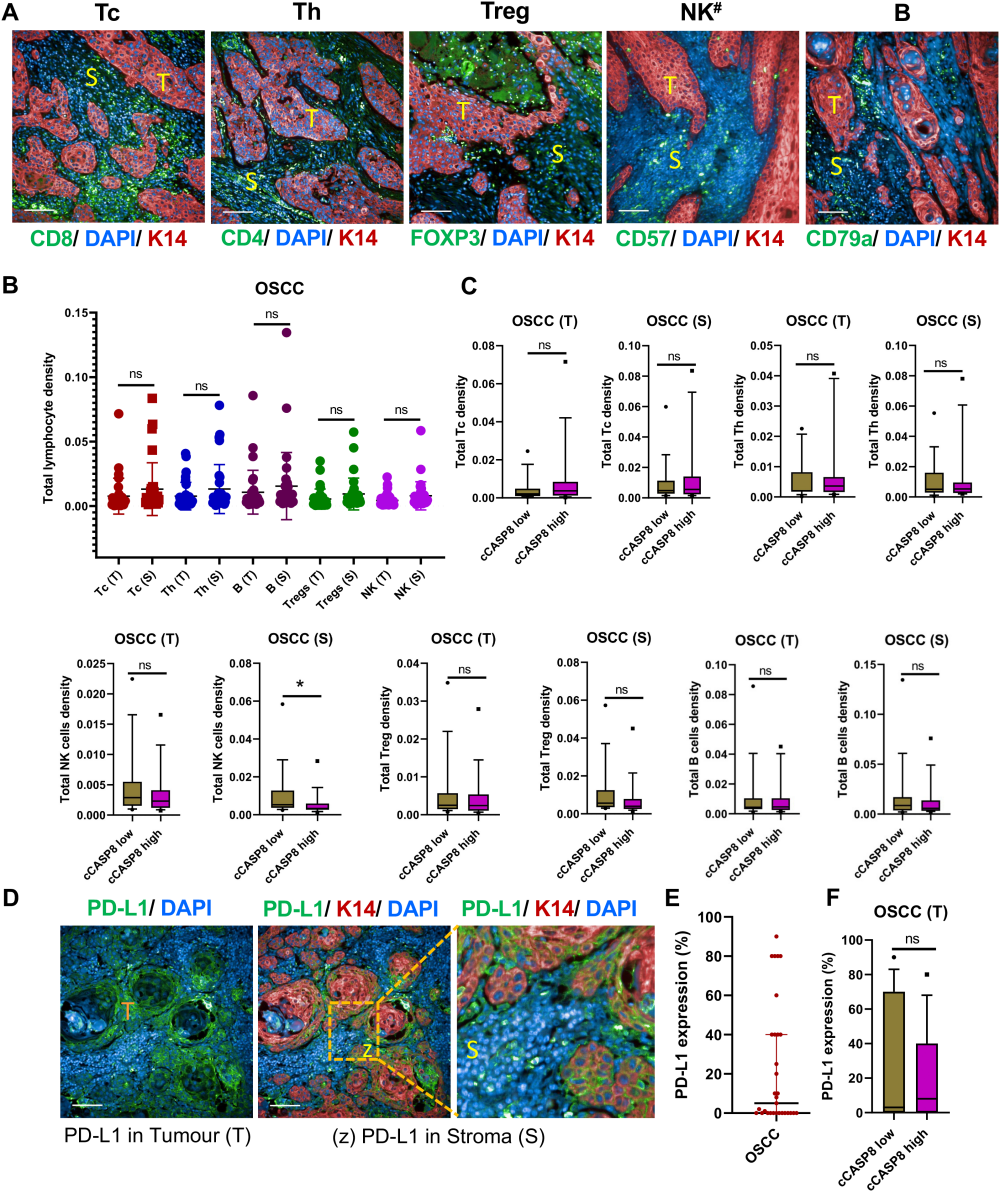
Correlation between apoptosis in tumour cells and TIL density. (A) Immunofluorescence staining for TILs (green) in OSCC sections labelled with anti‐keratin 14 (red) (representative images). ^#^CD57 labelling detects NK cells and a subset of lymphocytes. Note that the same tumour sections are shown in Figure 3A. (B) Dot plots representing total lymphocyte density enumerated by high content imaging analysis (HCA) pipeline. (C) Correlation between individual lymphocyte densities in tumour (T) or stroma (S) with cCASP8 expression. (D and E) Representative images of PD‐L1 expression in OSCC and its quantification. Note patchy membranous PD‐L1 staining in tumour islands (T) and lymphocytes (zoomed image, z). (F) Correlation between tumour PD‐L1 and cCASP8 expression. Data shown as mean ± SD. *p < 0.05; one‐way ANOVA with Šidák's multiple comparisons test. ns, not significant. Scale bars, 100 μm; *n* = 32.

We next examined lymphocyte density individually for Tc, Th, Treg, NK, and B cells (Figure 2A) in tumours with high or low levels of cCASP8 (based on median value). Digital whole‐slide analysis (supplementary material, Figures S2 and S3) showed no significant correlation between the presence of lymphocytes in tumour or stroma and CASP8 activation in tumour cells (Figure 2B,C).

We also examined expression of PD‐L1, which can protect tumours from lymphocyte‐mediated caspase activation (Figure 2D). There were very low levels of membranous PD‐L1 staining in most of the analysed tumours (0–5% of keratin 14‐positive area) (Figure 2E). A higher proportion of immune cells did express PD‐L1, but they were primarily located in the stroma (Figure 2D and supplementary material, Figure S6). There was no significant correlation between expression of PD‐L1 either by tumour or immune cells and caspase activation in tumours, and there was no survival benefit of low tumour PD‐L1 expression (HR: 1.476; 95% CI 0.561–3.886; *p* = 0.43) (Figure 2F and supplementary material, Figure S6 and Table S3).

### Apoptosis of TILs in stroma and tumour islands

**Figure 3:**
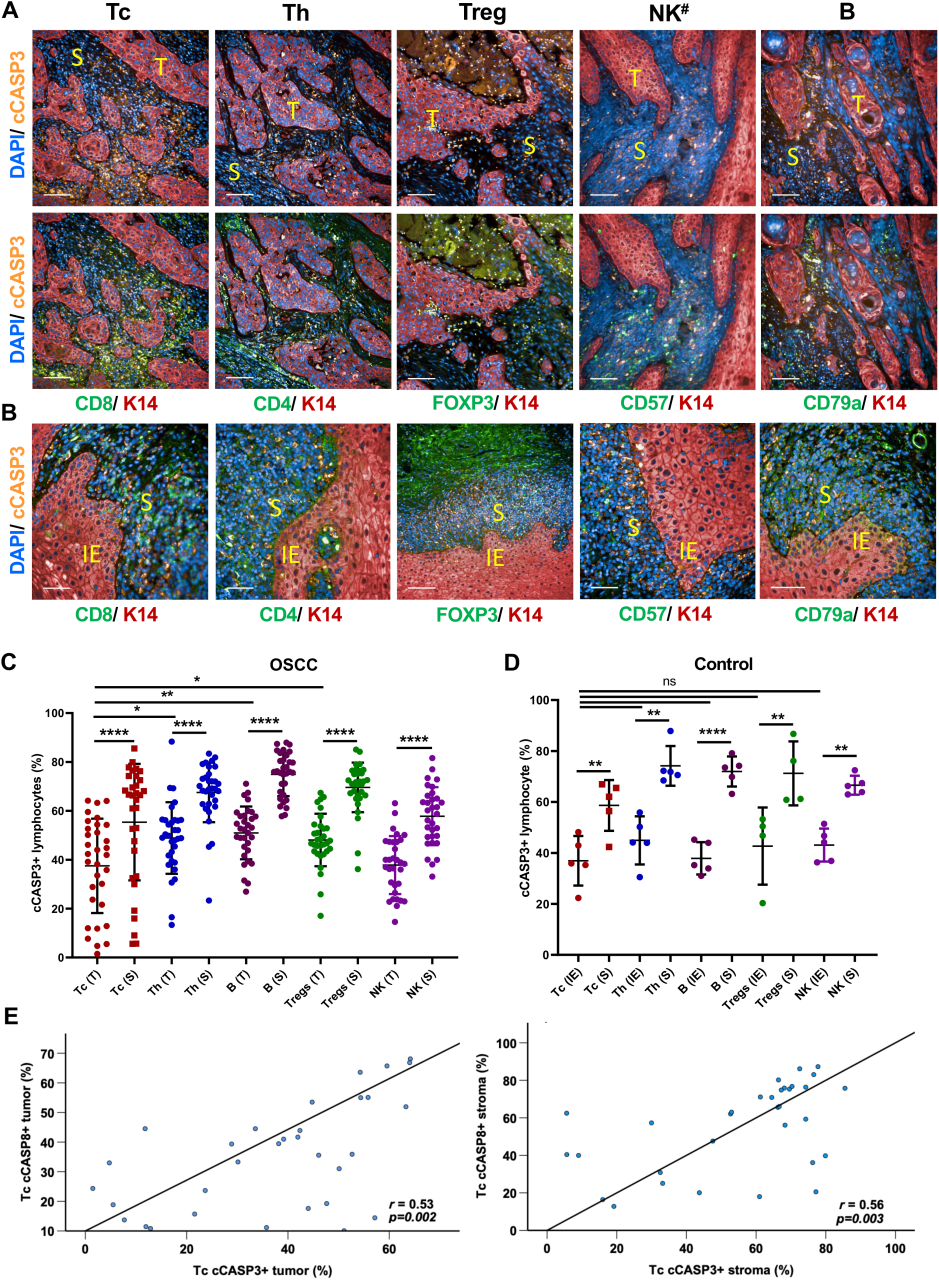
Apoptosis of TILs. Representative images of cCASP3 (orange) expression in lymphocytes (green) within tumour islands (T) and stroma (S) of OSCC stained with keratin 14 (red) (A) and in benign inflamed oral surface squamous epithelium control samples (IE and S regions indicated) (B) ^#^CD57 labelling detects NK cells and a subset of lymphocytes. Note that the same sections are shown in Figures 2A and 3A. (C and D) Dot plots representing percentage of lymphocytes with activated CASP3 in OSCC and controls. Tumour islands (T) and stroma (S) are shown separately in C (*n* = 32); IE and S are shown separately in D (*n* = 5). (E) Positive correlations observed between cCASP3 and cCASP8 activation in Tc in tumour epithelium (correlation coefficient *r* = 0.53) and stroma (*r* = 0.56). Data shown as mean ± SD. *p < 0.05, **p < 0.01 and ***p < 0.001, ****p < 0.0001; one‐way ANOVA with Šidák's multiple comparisons test. ns, not significant (C and D); Spearman correlation (E). Scale bars, 100 μm.

Multiplex immunofluorescence performed on OSCC sections showed relatively high levels of cCASP3 in all the lymphocyte subtypes examined (Tc, Th, Treg, NK, and B cells) (Figure 3A,C and supplementary material, Table S4). The proportion of lymphocytes labelling positive for cCASP3 was consistently higher in the stroma (60–76% positive; Figure 3C, and supplementary material, Table S4) compared to within the tumour islands (35–52%). This could be the result of activation‐induced cell death (AICD), suggesting that the stroma is more toxic to lymphocytes. The proportion of Tc with activated cCASP3 (cCASP3+Tc; 42 ± 19%) in tumour islands and in the stroma (66 ± 23%) was significantly lower than that of Th, Treg, and B cells (Figure 3C and supplementary material, Table S5). In the benign inflamed oral surface epithelium control samples used in optimisation, the level of cCASP3 activation in intraepithelial (IE) lymphocytes was relatively lower (36 ± 9.7%) than in tumour islands, while the control stromal lymphocytes had similar cCASP3 levels to those seen in tumour stroma (Figure 3B,D). However, no significant difference was observed between different lymphocyte subtypes (Figure 3D and supplementary material, Table S5).

We observed a significant positive correlation between cCASP8+Tc and cCASP3+Tc levels in both the stroma and tumour islands of OSCC (Figure 3E), consistent with CASP3 being activated downstream of CASP8 in the extrinsic pathway of apoptosis [12]. The strikingly higher activation of CASP3/8 in TILs than tumour epithelium could explain their inability to induce apoptosis in tumour cells even when TILs are present in abundance in tumour islands or in stroma (Figure 2C).

### FasL‐dependent apoptosis of Tc‐infiltrating tumour islands

**Figure 4:**
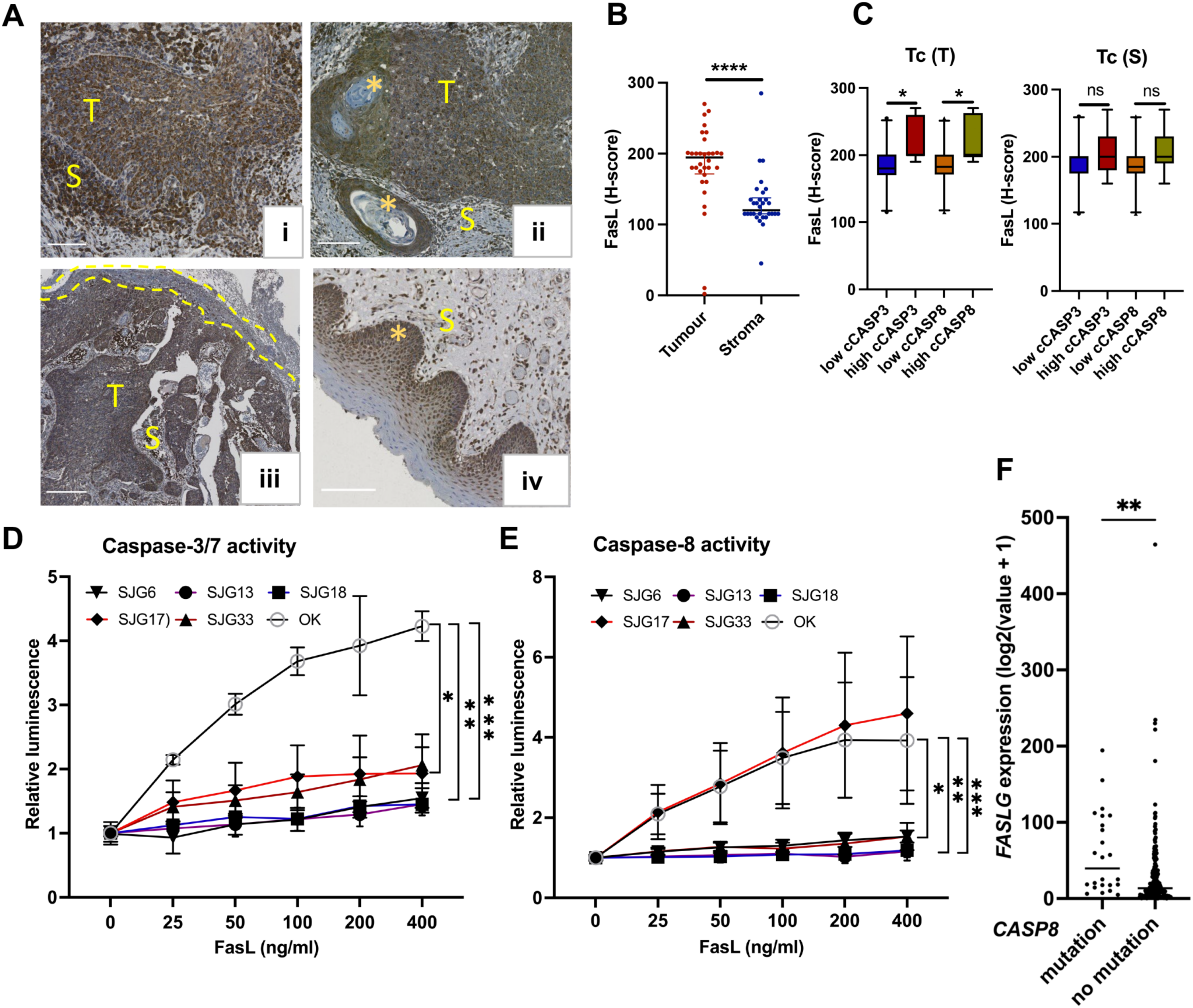
FasL‐mediated apoptosis of Tc in OSCC. (A) Representative IHC images showing high FasL expression in tumour islands (T) (i), loss of FasL expression in keratinised tumour islands (marked with *) (ii), loss of diffuse expression of FasL in dysplastic surface epithelium (yellow dotted line) compared to tumour (T) (iii), and a basal/lower prickle cell pattern of FasL expression in non‐dysplastic surface epithelium (marked by askterisk) (iv). Scale bars, 200 μm (*n* = 32). (B) Dot plots comparing FasL expression represented as H‐score in tumour islands and stroma. (C) Box plots representing correlation between FasL expression and high or low levels (based on median values) of cCASP3 and cCASP8 in Tc. (D and E) Caspase 3/7 and Caspase 8 activity in *CASP8* mutant (SJG6, SJG13, SJG18) versus *CASP8* wild type lines (OK, SJG17, SJG33) upon FasL treatment. (F) TCGA data showing higher mean *FASLG* expression in samples harbouring *CASP8* mutations. Data shown as mean ± SD (B, C, and F); *p < 0.05, **p < 0.01, and ***p < 0.001, ****p < 0.0001; Mann–Whitney test (B); multiple comparisons using ordinary one‐way ANOVA with the two‐stage linear step‐up procedure of Benjamini, Krieger, and Yekutieli (C); one‐way ANOVA with Tukey's multiple comparisons test, assays performed in triplicate and represented as mean ± SEM (D and E); unpaired *t*‐test (F). ns, not significant.

The FasL‐Fas‐Caspase8‐mediated apoptotic pathway plays a key role in maintaining immune cell homeostasis, and overexpression of FasL has been reported in various tumour types [19, 20, 21, 22]. We therefore examined tumour FasL expression in our sample set. Consistent with the literature, FasL immunohistochemical staining of OSCC sections (*n* = 32) showed low to very high FasL expression in the tumour islands and stroma (supplementary material, Figure S1C). Wherever present, a diffuse expression pattern for FasL was observed in tumour islands, with loss of expression in keratinised/well‐differentiated areas (*n* = 8) (Figure 4A,B) (supplementary material, Figure S1C). In non‐dysplastic surface epithelium (*n* = 22), FasL expression was limited to the basal and lower prickle cells (Figure 4A) with occasional nuclear staining, as previously observed in epidermal keratinocytes, fibroblasts, and T cells [23, 24]. There was more FasL expression in the tumour epithelium than the stroma (Figure 4B). Tumours with higher FasL expression (H‐score > 200) had a significantly higher proportion of cCASP3+Tc and cCASP8+Tc within the tumour islands but not in the stroma (Figure 4C). The prominent expression of FasL in tumour islands and, to a lesser extent, in the stroma provides a likely mechanism for induction of the extrinsic pathway of apoptosis in lymphocytes.

The low levels of cCASP3 and cCASP8 positivity in tumour cells (Figure 1A), despite the observed high FasL expression, indicates that OSCC keratinocytes are resistant to induction of the extrinsic pathway of apoptosis by FasL, consistent with earlier studies. To examine this further, we undertook FasL dose response experiments on OSCC cell lines of known *CASP8* mutation status and assessed CASP3 and CASP8 activation with the Caspase‐Glo assay. We found OSCC lines carrying missense (SJG6, SJG18) or nonsense (SJG13) mutations of *CASP8* had significantly lower levels of CASP3 and CASP8 activity compared to non‐malignant oral keratinocytes (Figure 4D,E; supplementary material, Table S6). The SJG33 OSCC line, which expresses wild type (WT) *CASP8*, also showed significantly reduced activation of both CASP3 and CASP8 compared to non‐malignant oral keratinocytes (OK).

The SJG17 OSCC line expresses WT *CASP8* and showed similar activation of CASP8 compared to non‐malignant OKs (Figure 4E and supplementary material, Table S6). Nevertheless, SJG17 showed reduced activation of CASP3 compared to non‐malignant OKs (Figure 4D and supplementary material, Table S6). One potential explanation is that SJG17 might express anti‐apoptotic proteins such as cIAP1. cIAPs are frequently overexpressed in oral cancers and are known to inhibit activation of caspase‐3, ‐7, and ‐9 but not of caspase‐8 [25, 26].

We next examined the *CASP8* mutation status of *FASLG* expressing tumours in The Cancer Genome Atlas (TCGA) head and neck squamous cell carcinoma (HNSCC) dataset using cBioPortal [27, 28]. *FASLG* is the gene encoding FASL. We observed that tumours with *CASP8* mutations had higher mean *FASLG* expression (Figure 4F), suggesting that the OSCC lines were resistant to FasL‐mediated apoptosis. *CASP8* mutations could potentially be a self‐defence mechanism in tumours whereby tumour cells resist apoptosis induction by neighbouring FasL overexpressing tumour cells or TILs.

### Higher apoptotic Tc within tumour islands are associated with the presence of metastatic disease

Activation of apoptosis in the TILs could have a major impact on the efficacy of immunomodulatory therapies. Recent studies identified the presence of terminally exhausted CD8+ T cells (Tex) in high frequencies in cancer patients, and these patients responded poorly to anti‐PD1 treatment [29]. When we looked at the single‐cell RNA sequencing data of Zheng *et al* [29] for CASP3 expression in Tex, we observed CASP3 positivity in 28% of the Tex cell population.

**Figure 5:**
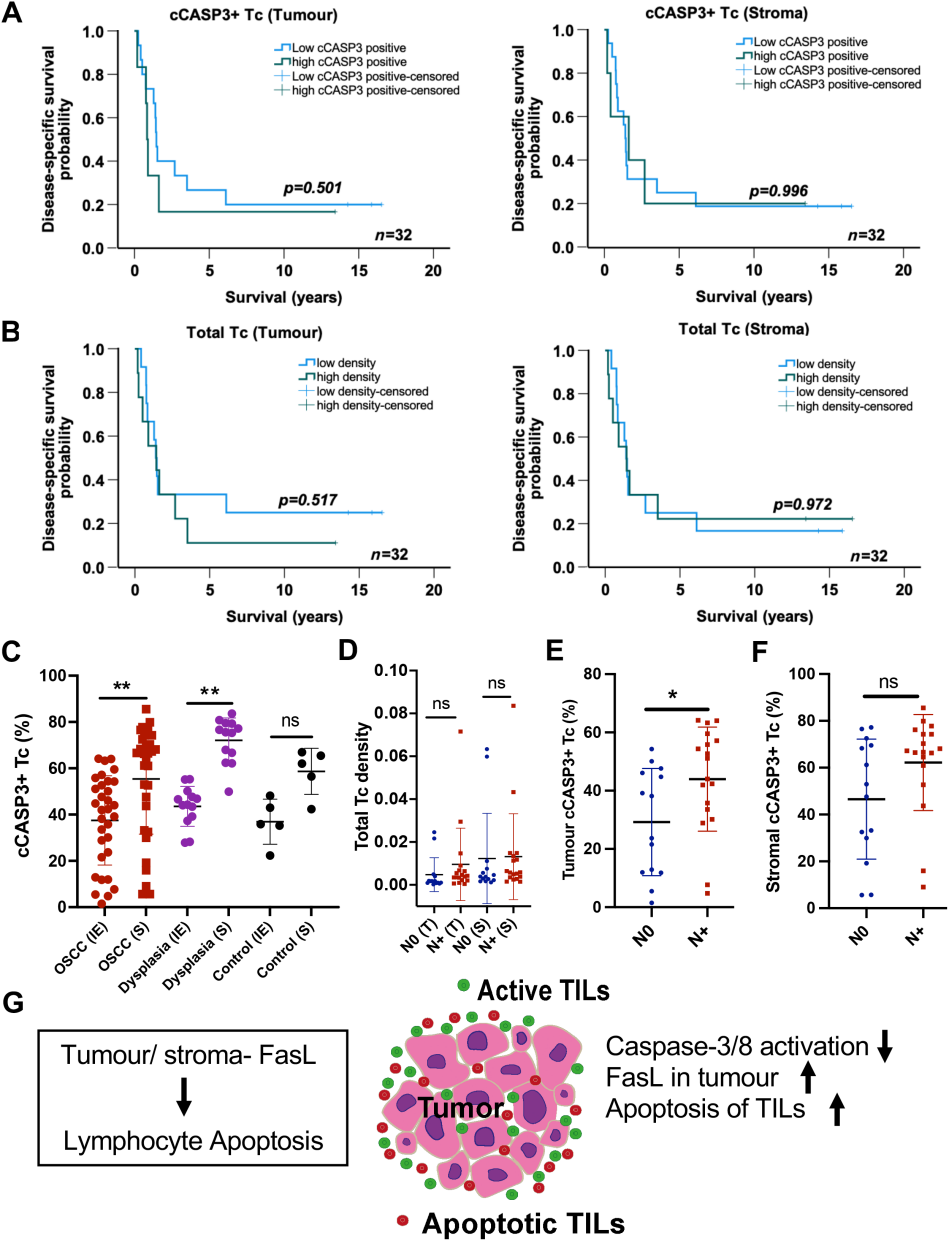
Apoptosis of Tc in OSCC patient samples. (A and B) Kaplan–Meier plots of DSS (log‐rank test), total Tc density, and cCASP3‐activated Tc in tumour epithelium and stroma (*n* = 32). (C) Dot plots representing percentage cCASP3+Tc in OSCC tumour islands (T) and stroma (S) (*n* = 32), dysplasias (IE and S)) (*n* = 13) and control oral mucosa (IE and S)) (*n* = 5). (D–F) Quantification of total Tc density and percentage of cCASP3+ Tc in tumour (T) and stroma (S) of lymph node metastatic OSCC patients. (G) Schematic describing a possible mechanism of tumour immune evasion in OSCC. Data shown as mean ± SD. *p < 0.05 and **p < 0.01, one‐sample *t*‐test and Wilcoxon signed‐rank test (C); one‐way ANOVA with Dunn's multiple comparisons test (D–F). ns, not significant.

We next examined the potential association between apoptotic (cCASP3+) Tc and clinical outcomes in our sample set. The relationship with DSS was assessed using the Cox proportional hazard model. We did not see a significant association between DSS and cCASP3+ Tc in the tumour epithelium (HR: 1.005; 95% CI 0.981–1.029; *p* = 0.683) or cCASP3+ Tc in the stroma (HR: 1.004; 95% CI 0.985–1.024; *p* = 0.674) (supplementary material, Table S7). The Kaplan–Meier curves for DSS did not show any significant correlation with cCASP3+ Tc [in tumour epithelium (*p* = 0.501) or stroma (*p* = 0.996)] or with total Tc density [in tumour epithelium (*p* = 0.517) or stroma (*p* = 0.972)] (Figure 5A,B).

We next compared the abundance of Tc in the epithelium and stroma of our OSCC, dysplasia, and control oral epithelium samples (Figure 5C). In OSCC and dysplasia there was a significantly higher proportion of apoptotic (cCASP3+) Tc within the stroma compared to the epithelium. Such a difference was not observed in control tissue or benign inflamed oral surface squamous epithelium and stroma (Figure 5C, and supplementary material, Table S9). Overall, the proportion of apoptotic Tc was greatest in OSCC, which also exhibited the highest variation between samples (supplementary material, Table S4). Dysplasias had 44 ± 8.6% apoptotic Tc in the intra‐epithelial region (IE) and 75 ± 9.6% in the stroma (S). In inflamed benign oral surface mucosa 35 ± 9.7% of Tc in the epithelium (IE) were apoptotic, compared with 62 ± 9.9% in the stroma (S), although statistical significance was not reached (Figure 5C and supplementary material, Table S9).

Finally, we examined the relationship with lymph‐node metastatic disease using multinominal regression analysis of the densities of subtyped TILs (total, positive for cCASP3 or negative for cCASP3) in the tumour stroma and within tumour epithelial islands of our OSCC samples (Figure 5D–F and supplementary material, Tables S7 and S8). There was a significantly higher proportion of apoptotic Tc in tumour islands, but not stroma, of patient samples with lymph node metastatic disease [odds ratio (OR): 1.046; 95%CI: 1.002–1.091; *p* = 0.039] (Figure 5E,F). This indicates the presence of a more aggressive tumour phenotype in patients with a higher proportion of apoptotic Tc.

## Discussion

We have shown using multiplex immunofluorescence that there are low levels of cCASP3 and cCASP8 in the epithelial compartment of human OSCC, irrespective of the degree of lymphocyte infiltration in the tumours. Activation of CASP8 and, to a lesser extent CASP3, was found in keratinised areas of the tumours. Previous studies showed a role for Caspase 8 in the terminal differentiation of epidermal keratinocytes [30] supporting the results presented here. Higher levels of epithelial cCASP3 were associated with better DSS in the total patient cohort. Higher levels of cCASP8 were associated with better DSS among the patients with lymph node metastases, suggesting that inactivating mutations in *CASP8* might be linked to a poorer clinical outcome.

As we did not observe a correlation between the densities of B, T, and NK cells with epithelial cCASP3/cCASP8 levels or with DSS, the reduced activation of cCASP8/3 could be due to other apoptosis‐resistance mechanisms [31, 32] rather than the levels of immune infiltration. Most previous studies of TIL infiltration in routine H&E‐stained sections of a variety of cancers showed a higher infiltrate to be prognostic of better survival, and TIL scores were used as an evaluation parameter in some immunotherapy trials [6, 33, 34]. Our failure to find a significant relationship between overall TIL density and DSS contrasts with some earlier studies [6, 8, 9, 10, 11] and could be because of our smaller sample size. The cohort we examined was normalised to high‐stage cases but was still heterogeneous with respect to multiple prognostic factors, including perineural invasion, excision margin status, vascular invasion, and comorbidities. Therefore, studies with larger cohorts are undoubtedly warranted.

Our multiplex immunofluorescence showed high levels of apoptotic B cells, Th cells, Treg cells, Tc cells, and NK cells that had infiltrated the tumour tissue. The findings are supported by an earlier demonstration of apoptotic lymphocytes in circulation and in oral tumours [35]. We were also able to analyse the clinical samples simultaneously for multiple mechanisms of acquired tumour resistance, including apoptosis of individual TIL subtypes, and evaluate their association with clinical outcome. To further investigate the mechanism driving induction of TIL apoptosis, we examined FasL levels by IHC and found high levels among stromal cells and in tumour islands. Tumour FasL expression was diffuse throughout OSCC tumour islands, except for loss of expression in keratinised areas, contrasting with non‐dysplastic surface epithelium, where FasL expression was restricted to basal and lower prickle cells.

The high expression of FasL in OSCC provides a potential mechanism for induction of apoptosis in TILs. Previous *in vitro* studies demonstrated apoptosis of OSCC cells co‐cultured with immortalised T lymphocytes in a Fas/FasL‐dependent manner. OSCC cell apoptosis can also be induced by incubation with FasL‐positive microvesicles derived from the plasma of patients with head and neck squamous cell carcinoma. Other studies showed high levels of apoptosis in the circulating T lymphocytes of patients with head and neck cancer compared to healthy control patients [35, 36]. Similar results are reported in additional cancer types [19, 22, 37, 38, 39, 40]. Recent studies also reported the presence of terminally exhausted Cytotoxic T lymphocytes (Tex), which are ineffective in eliciting an anti‐tumour response [29, 41]. The presence of a higher proportion of apoptotic or exhausted Tc in tumour islands suggests the potential toxicity of the tumour environment, which could affect the efficacy of immunomodulatory therapies.

The finding of high levels of FasL in OSCC raised the question of how OSCC epithelial cells evade apoptosis [13, 14, 15, 16, 17]. The *in vitro* studies performed here show OSCC lines with *CASP8* mutations were relatively more resistant to FasL‐induced apoptosis compared to those WT for *CASP8*. Co‐expression analysis of the TCGA HNSCC clinical dataset for *FASLG* expression in *CASP8* mutated samples supports our proposal that tumour cells are protected from FasL‐mediated apoptosis. Other studies have shown a similar correlation between *CASP8* mutations and *FASLG* expression in HNSCC tumours with a high level of immune infiltration [42, 43]. Reduced expression of the Fas receptor in OSCC compared to normal oral mucosa has also been reported [44] [36, 45, 46, 47]. Overall, these findings point to the existence of mechanisms that enable tumour epithelial cells to resist apoptosis regardless of the immune microenvironment.

When we examined the association of apoptotic TILs with clinical outcome, we found that a higher percentage of apoptotic Tc cells within tumour islands was correlated with the presence of lymph node metastases. The impaired function of lymphocytes might provide tumour cells with an additional survival advantage. Understanding how tumour cells can trigger apoptosis of TILs could improve the efficacy of cancer immunotherapies such as chimeric antigen receptor‐engineered T (CAR‐T) cells, TCR‐engineered T (TCR‐T) cells, and other immune therapies that currently result in poor clinical response in solid tumours [40, 41, 48, 49].

In conclusion, we have found that OSCC is resistant to TIL‐mediated activation of the extrinsic apoptotic pathway and, conversely, that OSCC can induce apoptosis in TILs (Figure 5G). A better understanding of apoptosis‐resistance mechanisms in tumours and identification of features of the microenvironment that could lead to apoptotic, exhausted, or dysfunctional T cells might shape the development of immunotherapy approaches with enhanced clinical efficacy.

## Author contributions statement

PGB and RAK performed wet lab experiments, analysed the data, and designed the experiments. RAK performed histopathological evaluations and OSCC sample procurement. FMW supervised the project. RAK, PGB and FMW prepared the manuscript.

## Supporting information


**Figure S1.** Representative images from control staining
**Figure S2.** Image analysis pipeline for cCASP3/8+ TILs in tumour and stroma
**Figure S3.** Image analysis pipeline for CASP3/8+ tumour region
**Figure S4.** Overview of Operetta analysis pipeline
**Figure S5.** Representative H&E images
**Figure S6.** PD‐L1 expression by immune cells
**Table S1.** List of antibodies used
**Table S2.** Cleaved caspase‐3 and ‐8 positivity in keratinised regions of OSCC and case‐wise details of cCASP‐3/‐8 expression in keratinised tumour regions
**Table S3.** Correlation between tumour (PD‐L1) and tumour (cCASP3+/cCASP8+)
**Table S4.** Descriptive statistics for TILs
**Table S5.** Multiple comparisons between cCASP3+ lymphocytes in OSCC (tumour and stroma) and control (IE and stroma)
**Table S6.** Caspase‐8 activation in CASP8 mutated or WT cells upon FasL (400 ng/ml) treatment and Caspase‐3/7 activation in CASP8 mutated or WT cells upon FasL (400 ng/ml) treatment
**Table S7.** COX regression analysis between tumour and TIL parameters with DSS
**Table S8.** Multinomial logistic regression analysis
**Table S9.** Multiple comparisons between cCASP3+Tc in OSCC (tumour and stroma), dysplasia (lE and stroma) and control (IE and stroma)

## Data Availability

The data generated in this study are available upon request from the corresponding authors.
