# Supplementary material for: Caspase activation in tumour‐infiltrating lymphocytes is associated with lymph node metastasis in oral squamous cell carcinoma
Source: J Pathol. 2023 Jul 13;261(1):43–54. doi: 10.1002/path.6145 (PMC10772935; doi:10.1002/path.6145)
Supplement: Supplementary file 1 — Figure S1. Representative images from control staining Figure S2. Image analysis pipeline for cCASP3/8+ TILs in tumour and stroma Figure S3. Image analysis pipeline for CASP3/8+ tumour region Figure S4. Overview of Operetta analysis pipeline Figure S5. Representative H&E images Figure S6. PD‐L1 expression by immune cells Table S1. List of antibodies used Table S2. Cleaved caspase‐3 and ‐8 positivity in keratinised regions of OSCC and case‐wise details of cCASP‐3/‐8 expression in keratinised tumour regions Table S3. Correlation between tumour (PD‐L1) and tumour (cCASP3+/cCASP8+) Table S4. Descriptive statistics for TILs Table S5. Multiple comparisons between cCASP3+ lymphocytes in OSCC (tumour and stroma) and control (IE and stroma) Table S6. Caspase‐8 activation in CASP8 mutated or WT cells upon FasL (400 ng/ml) treatment and Caspase‐3/7 activation in CASP8 mutated or WT cells upon FasL (400 ng/ml) treatment Table S7. COX regression analysis between tumour and TIL parameters with DSS Table S8. Multinomial logistic regression analysis Table S9. Multiple comparisons between cCASP3+Tc in OSCC (tumour and stroma), dysplasia (lE and stroma) and control (IE and stroma) [file PATH-261-43-s001.docx]

**Caspase activation in tumour-infiltrating lymphocytes is associated with lymph node metastasis in oral squamous cell carcinoma**

PG Bhosale, RA Kennedy *et al. J Pathol* <https://doi.org/10.1002/path.6145>

**Supplementary Figures S1–S6**


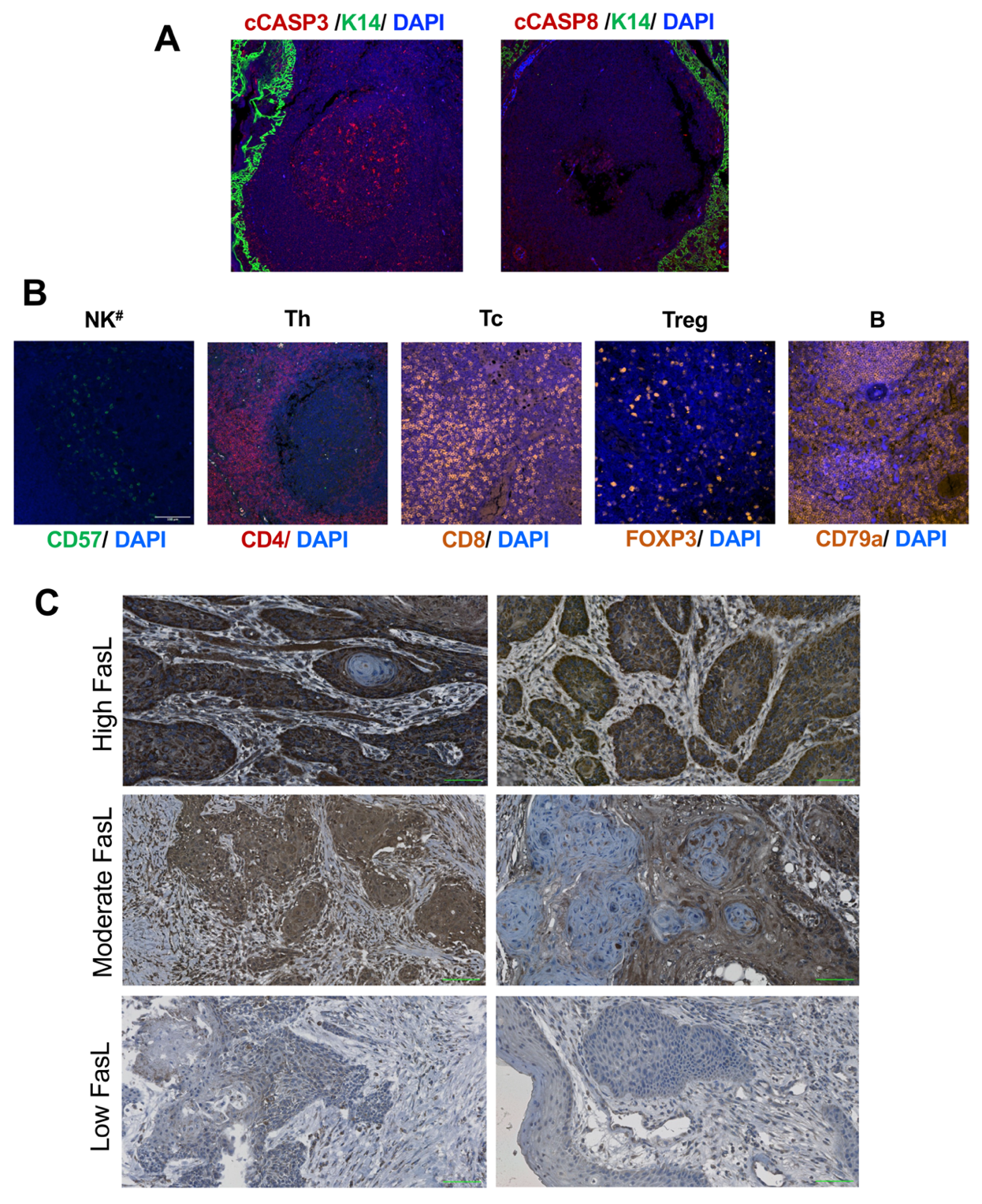


**Figure S1 Representative images from control staining.** Tonsil or lymph node-positive control staining. (A) Tonsil sections stained with cCASP3/ cCASP8 (very low activation) showing staining in germinal centre (*). (B) Staining for NK, ^#^Subset of T cells expressing CD57, Th, Tc, Treg, and B cells in tonsil or lymph-node sections. (C) Representative IHC images showing high, moderate, low/no FasL staining in tumour island and stroma of OSCCs.

**Figure S2. Image analysis pipeline for cCASP3/8+ TILs in tumour and stroma.** Step-by-step high-content image analysis pipeline generated via Harmony software to identify cCASP3/8+ TILs in tumour and stroma.

**Analysis sequence: multiplex_TILs_cCASP3_tumour_stroma"**

Page 1 of 4

**Calculate Input Method Output Morphology**

**Properties (2) Population :** Tumour **Method :** Standard Property Prefix : Region based on K14 Area Stroma (global)

(global) Morphology

**Region :** Stroma

**Calculate Input Method Output Morphology**

**Properties**

**Population :** Tumour **Method :** Standard Property Prefix : Region based on K14 Area Tumour Region

(global) (global) Morphology

**Region :** Tumour Region based on K14 (global)

**Select Region Input Method Output**

**Population :** Tumour **Method :** Resize Output Region :

Region based on K14 Region [%] Stroma (global) Outer Border : -100 %

**Region :** Tumour Region Outer Population :

based on K14 (global) Tumour Region based on

K14 (global)

Outer Region : Tumour Region based on K14 (global)

Inner Border : 0 %

**Find Image Input Method Output Region**

**Channel :** Alexa 647 **Method :** Common Output Population : (global) Threshold Tumour Region based **ROI :** Imaged Area Threshold : 0.4 on K14 (global) (global) Split into Objects Output Region : **ROI Region :** Image Area : > 200 px² Tumour Region based Area on K14 (global)

**Input Image Input**

**Flatfield Correction :** Basic Brightfield Correction

**Stack Processing :** Maximum Projection Create Global Image

**Min. Global Binning :** Dynamic

**Figure S2 (Continued)**

Page 2 of 4

**Find Spots (4) Input Method Output**

**Channel :** Alexa 555 **Method :** A Output Population : (global) Relative Spot Spots Alexa555

Intensity : > 0.03 (cCASP3+

**ROI :** Tumour Region Splitting Sensitivity : 1 Lymphocytes) based on K14 (global) Calculate Spot (Stroma) **ROI Region :** Stroma Properties

**Find Spots (3) Input Method Output**

**Channel :** Alexa 488 **Method :** A Output Population : (global) Relative Spot Spots Alexa488 **ROI :** Tumour Region Intensity : > 0.03 (lymphocytes) based on K14 (global) Splitting Sensitivity : 1 (Stroma)

**ROI Region :** Stroma Calculate Spot

Properties

**Select Input Method Output Population**

**Population :** Spots **Method :** Select by Output Population : Alexa488 (lymphocyte) Mask single posi Spots (Tumour) Region : Spot Alexa488 (cCASP3-

Mask Population : lymphocyte)

Spots Alexa555 (Tumour) cCASP3+ lymphocyte

(Tumour)

Mask Region : Spot Select by : Geometrical Center

Use Inverted Mask

**Find Spots (2) Input Method Output**

**Channel :** Alexa 555 **Method :** A Output Population : (global) Relative Spot Spots Alexa555 **ROI :** Tumour Region Intensity : > 0.03 cCASP3+

based on K14 (global) Splitting Sensitivity : 1 lymphocyte (Tumour)

**ROI Region :** Spots Calculate Spot

Properties

**Find Spots Input Method Output**

**Channel :** Alexa 488 **Method :** A Output Population : (global) Relative Spot Spots Alexa488 **ROI :** Tumour Region Intensity : > 0.03 (lymphocyte) based on K14 (global) Splitting Sensitivity : 1 (Tumour)

**ROI Region :** Tumour Calculate Spot Region based on K14 Properties (global)

**Figure S2 (Continued)**

Page 3 of 4

**Define Results Results**

**Method :** List of Outputs

**Population : Spots Alexa555 cCASP3+ lymphocyte (Tumour)**

Number of Objects

**Population : Spots Alexa488 (lymphocyte) (Tumour)**

Number of Objects

**Population : single posi Spots Alexa488 (cCASP3- lymphocyte) (Tumour)**

Number of Objects

**Population : Spots Alexa555 (cCASP3+ Lymphocytes) (Stroma)**

Number of Objects

**Population : Spots Alexa488 (lymphocytes) (Stroma)**

Number of Objects

**Population : Tumour Region based on K14 (global)**

Number of Objects

**Population : single positive Alexa488 (cCASP3- Lymphocytes) (Stroma)**

Number of Objects

**Method :** Standard Output

Tumour Region based on K14 (global) - Tumour Region (global) Morphology Area [µm²] : Mean

Output Name : Tumour Region based on K14 (global) - Tumour Region

(global) Morphology Area [µm²] - Mean per Well

**Method :** Formula Output Formula : a/b

Population Type : Objects

**Select Input Method Output Population (2)**

**Population :** Spots **Method :** Select by Output Population : Alexa488 Mask single positive (lymphocytes) Region : Spot Alexa488 (cCASP3- (Stroma) Mask Population : Lymphocytes)

Spots Alexa555 (Stroma) (cCASP3+

Lymphocytes) (Stroma) Mask Region : Spot Select by : Geometrical Center

Use Inverted Mask

**Figure S2 (Continued)**

Page 4 of 4

Variable a : single positive Alexa488 (cCASP3- Lymphocytes) (Stroma) - Spot Area [px²] Sum

Variable b : Tumour Region based on K14 (global) - Stroma (global)

Morphology Area [µm²] Sum

Output Name : singlealexa488spots per Area Density (Stroma)

**Method :** Standard Output

Tumour Region based on K14 (global) - Stroma (global) Morphology Area [µm²] : Mean

Output Name : Stroma (global) Morphology Area [µm²] - Mean per Well

**Method :** Formula Output Formula : a/b

Population Type : Objects

Variable a : Spots Alexa488 (lymphocyte) (Tumour) - Spot Area [px²] Sum Variable b : Tumour Region based on K14 (global) - Tumour Region (global) Morphology Area [µm²] Sum

Output Name : total 488 spot density_tumour_region

**Method :** Formula Output Formula : a/b

Population Type : Objects

Variable a : Spots Alexa488 (lymphocytes) (Stroma) - Spot Area [px²] Sum

Variable b : Tumour Region based on K14 (global) - Stroma (global) Morphology Area [µm²] Sum

Output Name : total 488 spot density_stroma_region

**Method :** Formula Output Formula : a/b

Population Type : Objects

Variable a : Spots Alexa555 cCASP3+ lymphocyte (Tumour) - Spot Area [px²] Sum

Variable b : Tumour Region based on K14 (global) - Tumour Region (global)

Morphology Area [µm²] Sum

Output Name : 555_spot density_tumour

**Method :** Formula Output Formula : a/b

Population Type : Objects

Variable a : Spots Alexa555 (cCASP3+ Lymphocytes) (Stroma) - Spot Area [px²] Sum

Variable b : Tumour Region based on K14 (global) - Stroma (global) Morphology Area [µm²] Sum

Output Name : 555_spot density_stroma

**Object Results**

Population : Spots Alexa555 cCASP3+ lymphocyte (Tumour) : None Population : Spots Alexa488 (lymphocyte) (Tumour) : None

Population : single posi Spots Alexa488 (cCASP3- lymphocyte) (Tumour) : None

Population : Spots Alexa555 (cCASP3+ Lymphocytes) (Stroma) : None

Population : Spots Alexa488 (lymphocytes) (Stroma) : None Population : Tumour Region based on K14 (global) : None

Population : single positive Alexa488 (cCASP3- Lymphocytes) (Stroma) : None

**Figure S3. Image analysis pipeline for CASP+ tumour region.** Step-by-step high-content image analysis pipeline generated via Harmony software to identify cCASP3/8+ tumour region.

**Analysis Sequence "cCASP3/8_analysis_tumour"**

Page 1 of 4

**Find Spots (2) Input Method Output**

**Channel :** Alexa 555 **Method :** A Output Population : (global) Relative Spot Spots Alexa555 **ROI :** Tumour Region Intensity : > 0.03 (Tumour)

(global) Splitting Sensitivity : 1

**ROI Region :** Tumour Calculate Spot Region (global) Properties

**Find Spots Input Method Output**

**Channel :** Alexa 488 **Method :** A Output Population : (global) Relative Spot Spots Alexa488 **ROI :** Tumour Region Intensity : > 0.03 (Tumour)

(global) Splitting Sensitivity : 1

**ROI Region :** Tumour Calculate Spot Region (global) Properties

**Calculate Input Method Output Morphology**

**Properties (2)**

**Population :** Tumour **Method :** Standard Property Prefix : Region (global) Area Stroma (global)

**Region :** Tumour Region Morphology

(global)

**Calculate Input Method Output Morphology**

**Properties**

**Population :** Tumour **Method :** Standard Property Prefix : Region (global) Area Tumour Region

**Region :** Tumour Region (global) Morphology

(global)

**Find Image Input Method Output Region**

**Channel :** Alexa 647 **Method :** Common Output Population : (global) Threshold Tumour Region

**ROI :** Imaged Area Threshold : 0.4 (global)

(global) Split into Objects Output Region : **ROI Region :** Imaged Area : > 200 px² Tumour Region Area (global)

**Input Image Input**

**Flatfield Correction :** Basic

**Stack Processing :** Maximum Projection Create Global Image

**Min. Global Binning :** Dynamic

**Figure S3 (Continued)**

Page 2 of 4

**Find Image Input Method Output Region (2)**

**Channel :** Alexa 647 **Method :** Common Output Population : (global) Threshold Tumour region

**ROI :** Imaged Area Threshold : 0.4 overall

(global) Area : > 200 px² Output Region :

**ROI Region :** Imaged Fill Holes Tumour region

Area overall

**Calculate Input Method Output Intensity**

**Properties (2)**

**Channel :** Alexa 555 **Method :** Standard Property Prefix : (global) Mean Intensity excluded

**Population :** Tumour lymphocytes Alexa

Region (global) 555 (global)

**Region :** excluded lymphocytes

**Select Region Input Method Output**

**Population :** Tumour **Method :** Restrict by Output Region : Region (global) Mask excluded **Region :** Tumour Region Population : Tumour lymphocytes (global) Region (global)

Mask Region : Spots Use Inverted Mask

**Calculate Input Method Output Intensity**

**Properties**

**Channel :** Alexa 555 **Method :** Standard Property Prefix : (global) Mean Intensity Spot Alexa

**Population :** Spots 555 (global)

Alexa555 (Tumour) Selected

**Region :** Spot

**Select Input Method Output Population (3)**

**Population :** Spots **Method :** Select by Output Population : Alexa555 (Tumour) Mask Spots Alexa555

Region : Spot (Tumour) Selected

Mask Population : Spots Alexa488 (Tumour)

Mask Region : Spot Select by : Geometrical Center

Use Inverted Mask

**Figure S3 (Continued)**

Page 3 of 4

**Calculate Input Method Output Morphology**

**Properties (4)**

**Population :** Tumour **Method :** Standard Property Prefix : Region (global) Area Tumour Region

**Region :** Tumour Region (global)

(global)

**Calculate Input Method Output Morphology**

**Properties (3)**

**Population :** Tumour **Method :** Standard Property Prefix : Alexa555+ Region Area Tumour Region Area

(excluded lymphocytes) (excluded

lymphocytes)

**Region :** Tumour Morphology Area

Alexa555+ Region (excluded lymphocytes)

**Find Image Input Method Output Region (3)**

**Channel :** Alexa 555 **Method :** Absolute Output Population : (global) Threshold Tumour Alexa555+ **ROI :** Tumour Region Lowest Intensity : ≥ Region (excluded (global) 1300 lymphocytes)

**ROI Region :** excluded Highest Intensity : ≤ Output Region : lymphocytes INF Tumour Alexa555+

Split into Objects Region (excluded

Area : > 0 px² lymphocytes)

**Calculate Input Method Output Intensity**

**Properties (3)**

**Channel :** Alexa 555 **Method :** Standard Property Prefix : (global) Mean Intensity Tumour

**Population :** Tumour region overall

region overall without lymphcytes

**Region :** Tumour Alexa 555 (global)

region overall without lymphcytes

**Select Region Input Method Output (2)**

**Population :** Tumour **Method :** Restrict by Output Region : region overall Mask Tumour region **Region :** Tumour Population : Spots overall without region overall Alexa488 (Tumour) lymphcytes

Mask Region : Spot Use Inverted Mask

**Figure S3 (Continued)**

Page 4 of 4

**Define Results**

**Results**

**Method :** List of Outputs

**Population : Spots Alexa488 (Tumour)**

Spot Area [px²] : Sum

**Population : Spots Alexa555 (Tumour)**

Spot Area [px²] : Sum

**Population : Spots Alexa555 (Tumour) Selected**

Number of Objects

**Population : Tumour Region (global)**

Number of Objects

**Method :** Standard Output

Tumour Region (global) - Intensity excluded lymphocytes Alexa 555 (global) Mean : Mean

Output Name : Tumour Region (global) - Intensity excluded lymphocytes Alexa 555 (global) Mean - Mean per Well

**Method :** Standard Output

Tumour region overall - Intensity Tumour region overall without lymphcytes Alexa 555 (global) Mean : Mean

Output Name : Tumour region overall - Intensity Tumour region overall without lymphcytes Alexa 555 (global) Mean - Mean per Well

**Method :** Formula Output Formula : a/b*100 Population Type : Objects

Variable a : Tumour Alexa555+ Region (excluded lymphocytes) - Tumour

Region Area (excluded lymphocytes) Morphology Area Area [µm²] Mean Variable b : Tumour Region (global) - Tumour Region (global) Area [µm²] Mean

Output Name : % Tumour 555+ Region (excluded lymphocytes) in each Tumour

**Method :** Standard Output

Tumour Alexa555+ Region (excluded lymphocytes) - Tumour Region Area (excluded lymphocytes) Morphology Area Area [µm²] : Mean

Output Name : Tumour Alexa555+ Region (excluded lymphocytes) - Tumour Region Area (excluded lymphocytes) Morphology Area Area [µm²] - Mean per Well

**Method :** Formula Output Formula : (a/b)*100 Population Type : Objects

Variable a : Tumour Alexa555+ Region (excluded lymphocytes) - Tumour Region Area (excluded lymphocytes) Morphology Area Area [µm²] Sum Variable b : Tumour Region (global) - Tumour Region (global) Morphology Area [µm²] Sum

Output Name : % casp8+ tumour area

**Object Results**

Population : Spots Alexa488 (Tumour) : None Population : Spots Alexa555 (Tumour) : None Population : Spots Alexa555 (Tumour) Selected : None Population : Tumour region overall : None

Population : Tumour Alexa555+ Region (excluded lymphocytes) : ALL Population : Tumour Region (global) : ALL


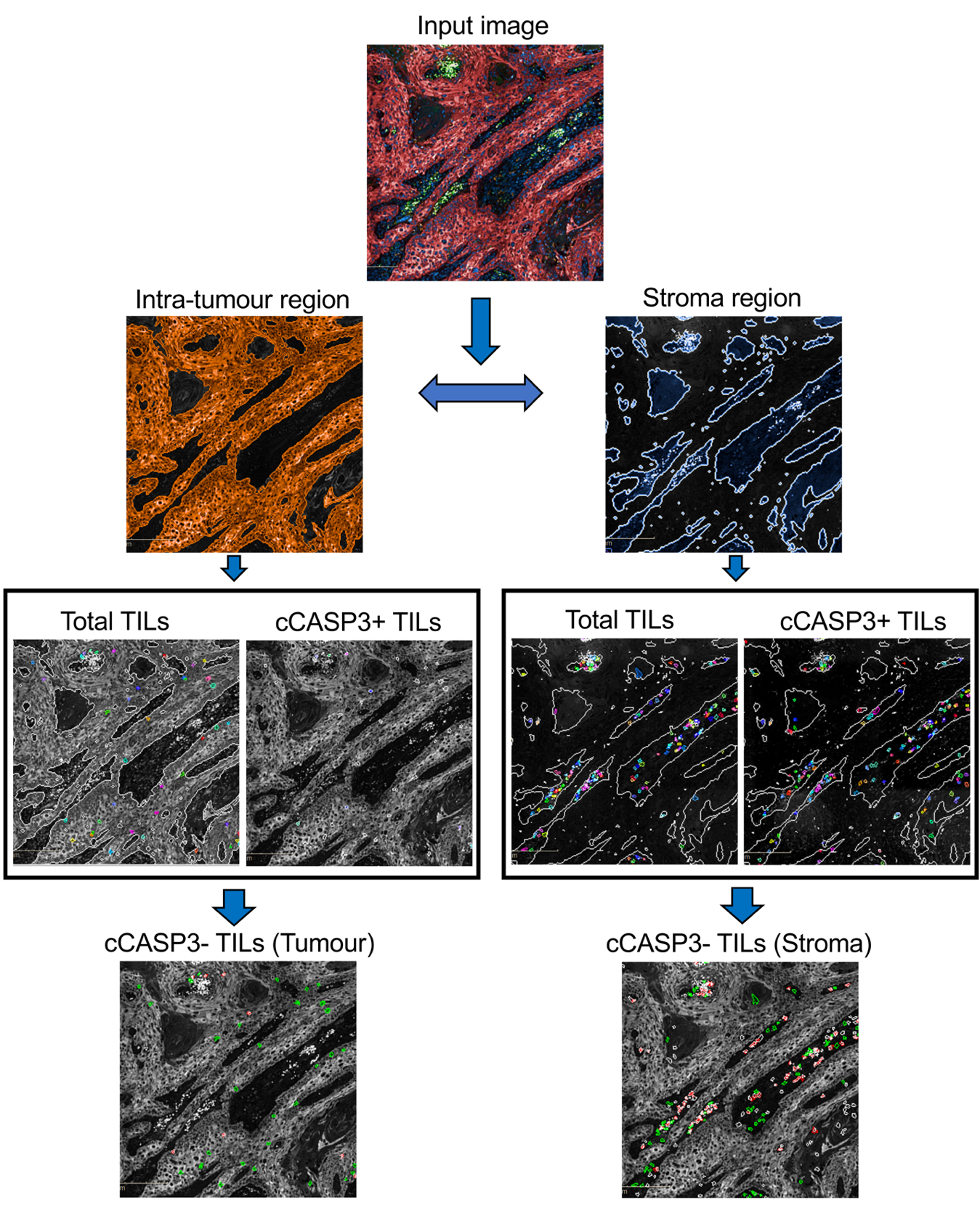


**Figure S4. Overview of Operetta analysis pipeline.** Flowchart demonstrating overview of high-content image analysis pipeline (Operetta CLS-HCA analysis using Harmony software) to identify cCASP3/8+ TILs in tumour and stroma.


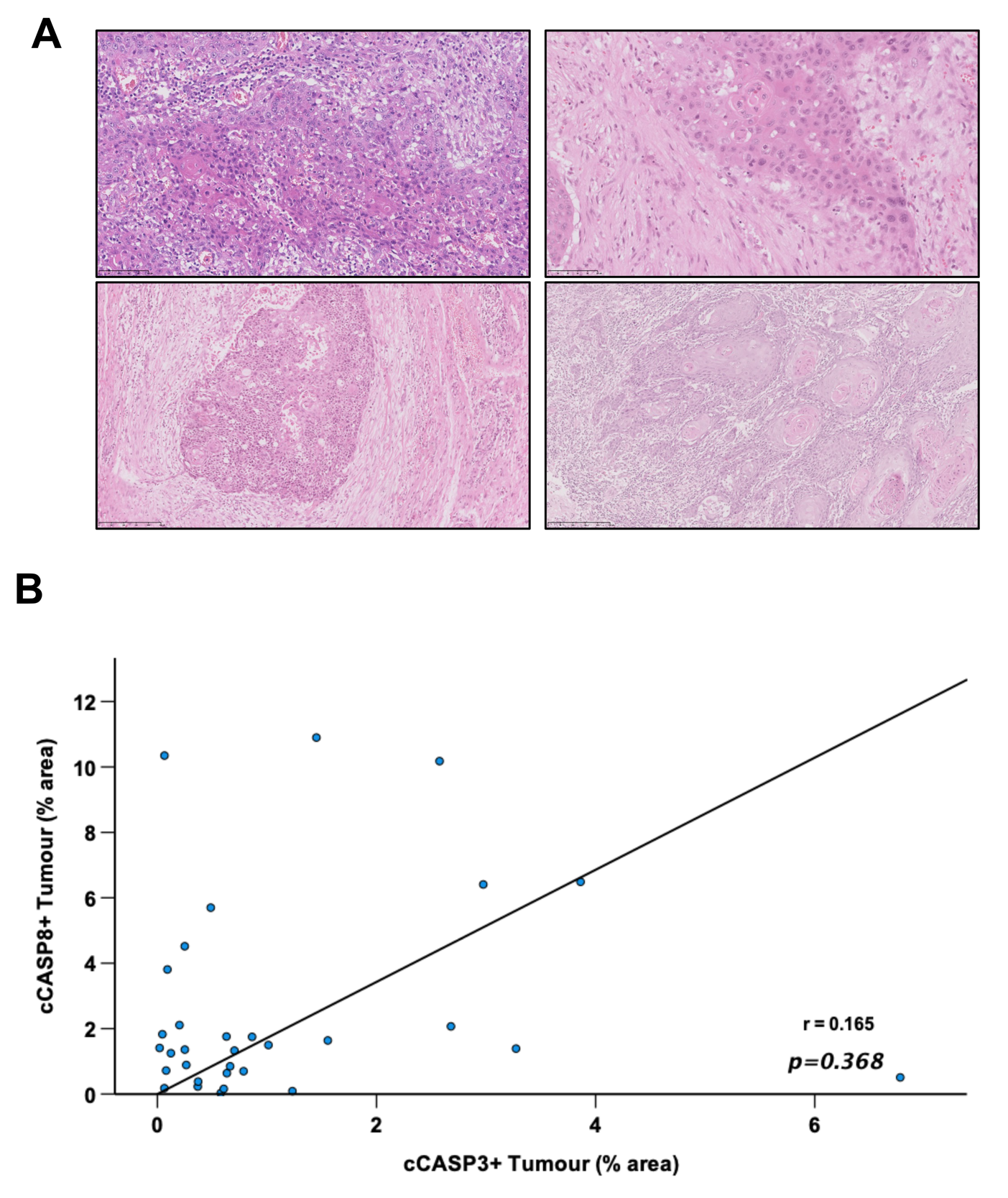


**Figure S5. Representative H&E images.** (A) Representative H&E-stained OSCC sections demonstrating different degrees of tumour-infiltrating lymphocytes evaluated by pathologist. (B) Correlation plot depicting cCASP3+ and cCASP8+ in tumour areas.


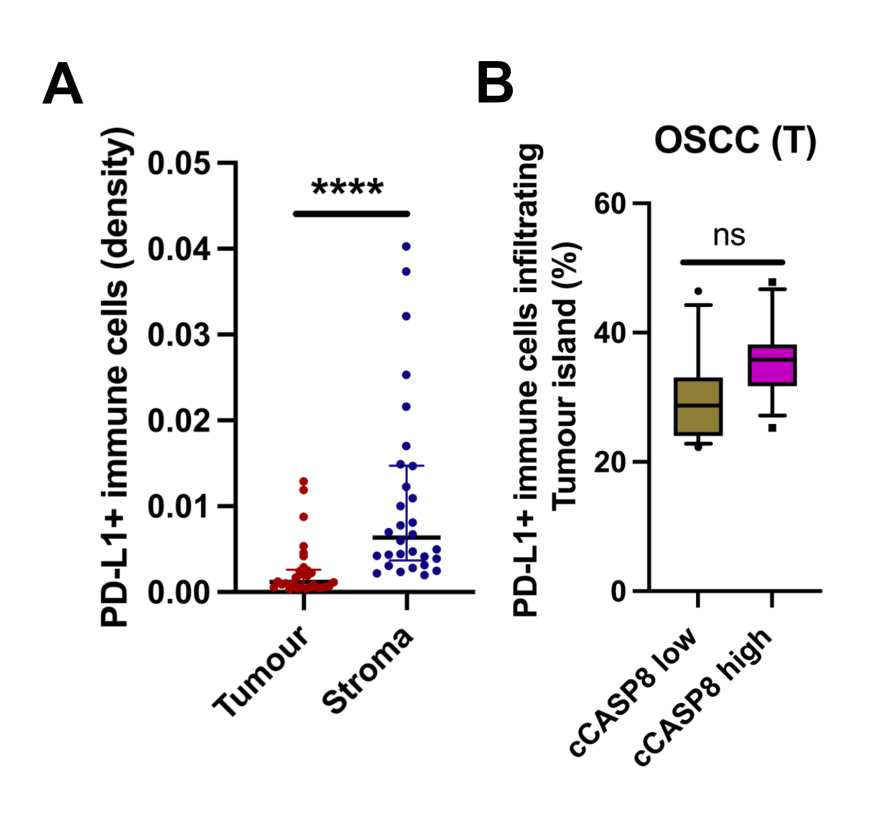


**Figure S6. PD-L1 expression by immune cells.** (A) PD-L1 expressing immune cells in tumour island (T) and in stroma. (B) No difference in Casp8 activation in tumour cells depending on PD-L1 positivity of tumour infiltrating immune cells. Data shown as mean ± SD. *****p* < 0.0001; one-way ANOVA with Šidák’s multiple comparisons test. ns, not significant. *n* = 32

**Supplementary Tables S1–S9**

**Table S1. Antibodies used**

|  | **Antibody** | **Company** | **Catalogue** | **Dilution** | **Antigen retrieval solution** |
| --- | --- | --- | --- | --- | --- |
| 1 | Cleaved Caspase-8 (Asp391) (18C8) | Cell Signaling Technology, Danvers, MA, USA | 9496S | 1/100 | Citrate pH6 |
| 2 | Cleaved Caspase-3 (Asp175) (5A1E) | Cell Signaling Technology | 9664 | 1/200 | Citrate pH6 |
| 3 | CD4 (4B12) | Leica Biosystems, Wetzlar, Germany | NCL-L-CD4-368 | 1/100 | TE pH9 |
| 4 | CD45 (X16/99) | Leica Biosystems | LCA-L-CE | 1/40 | Citrate pH6 |
| 5 | CD57 (NK-1) | Leica Biosystems | PA0443 | Ready to use | Citrate pH6 |
| 6 | CD8 alpha (C8/468) | Novus Biologicals, Englewood, CO, USA | NBP2-32952 | 1/200 | Citrate pH6 |
| 7 | CD79A (HM57) | Novus Biologicals | NB100-64347 | 1/100 | Citrate pH6 |
| 8 | FoxP3 Clone (259D/C7) | BD Pharmingen™ (BD Biosciences), Franklin Lakes, NJ, USA | 560044 | 1/100 | Citrate pH6 |
| 9 | Fas Ligand | Abcam, Cambridge, UK | ab134401 | 1/300 | Citrate pH6 |
| 10 | PD-L1 (7310) Bond RTU | Leica Biosystems | PA0832 | Ready to use | Citrate pH6 |
| 11 | Keratin 14 | Biolegend, San Diego, CA, USA (Formerly Covance SIG-3476-100) | 906001 (discontinued) alternate (906004) | 1/1000 | N/A |
| 12 | Goat anti-Chicken IgY (H+L) Secondary Antibody, Alexa Fluor 647 | Thermo Fisher Scientific, Waltham, MA, USA | A21449 | 1/300 | N/A |
| 13 | Goat anti-Rabbit IgG (H+L) Secondary Antibody, Alexa Fluor 555 | Thermo Fisher Scientific | A21428 | 1/300 | N/A |
| 14 | Goat anti-Mouse IgG (H+L) Secondary Antibody, Alexa Fluor Plus 488 | Thermo Fisher Scientific | A32723 | 1/300 | N/A |

**Table S2. Cleaved caspase-3 and -8 positivity in keratinised regions of OSCC and case-wise details of cCASP-3/-8 expression in keratinised tumour regions.**

|  | **Number of cases/ total cases** | **%** |
| --- | --- | --- |
| **Keratinised tumours** | 18/32 | 56.3 |
| **cCASP3 active in keratinised areas** | 6/18 (all 6 positive for cCASP8) | 33.3 |
| **cCASP8 active in keratinised areas** | 17/18 | 94.4 |

|  | **Keratinised tumours (*n* = 18)** | |
| --- | --- | --- |
| **Case No.** | **cCASP3** | **cCASP8** |
| 1 | 0 | 1 |
| 2 | 0 | 1 |
| 3 | 0 | 1 |
| 4 | 1 | 1 |
| 5 | 0 | 1 |
| 6 | 0 | 0 |
| 7 | 1 | 1 |
| 8 | 1 | 1 |
| 9 | 0 | 1 |
| 10 | 0 | 1 |
| 11 | 1 | 1 |
| 12 | 0 | 1 |
| 13 | 1 | 1 |
| 14 | 0 | 1 |
| 15 | 1 | 1 |
| 16 | 0 | 1 |
| 17 | 0 | 1 |
| 18 | 0 | 1 |

**Table S3. Correlation between tumour (PD-L1) and tumour (cCASP3+/cCASP8+).**

|  | **Tumour PD-L1** | | |
| --- | --- | --- | --- |
|  | **Pearson's R Value** | ***p*-value summary** | ***p*-value** |
| **Tumour CASP8+** | –0.17 | ns | 0.654 |
| **Tumour CASP3+** | 0.30 | ns | 0.103 |
| ns: not significant |  |  |  |

**Table S4. Descriptive statistics for TILs.**

|  | **Mean** | **Median** | **SD** | **Range** | **Minimum** | **Maximum** | **Percentiles** | | |
| --- | --- | --- | --- | --- | --- | --- | --- | --- | --- |
|  |  |  |  |  |  |  | **25** | **50** | **75** |
| **cCASP8+ tumour %** | 2.5982 | 1.4000 | 3.1105 | 10.8700 | 0.0300 | 10.9000 | 0.6550 | 1.4000 | 3.3850 |
| **cCASP3+ tumour %** | 1.1114 | 0.6186 | 1.4640 | 6.7600 | 0.0200 | 6.7800 | 0.2141 | 0.6186 | 1.3973 |
| **PD-L1 tumour %** | 22.9355 | 5.0000 | 30.9364 | 90.0000 | 0.0000 | 90.0000 | 0.0000 | 5.0000 | 40.0000 |
| **FasL tumour (H-score)** | 194.3667 | 199.5000 | 35.8642 | 155.0000 | 115.0000 | 270.0000 | 178.7500 | 199.5000 | 205.7500 |
| **FasL stroma (H-score)** | 131.1667 | 120.0000 | 39.4725 | 240.0000 | 45.0000 | 285.0000 | 115.0000 | 120.0000 | 137.5000 |
| **Tc density (tumour)** | 0.0075 | 0.0034 | 0.0137 | 0.0713 | 0.0002 | 0.0715 | 0.0010 | 0.0034 | 0.0061 |
| **Tc density (stroma)** | 0.0128 | 0.0051 | 0.0202 | 0.0824 | 0.0012 | 0.0836 | 0.0027 | 0.0051 | 0.0116 |
| **NK cells density (tumour)** | 0.0043 | 0.0026 | 0.0049 | 0.0217 | 0.0007 | 0.0225 | 0.0014 | 0.0026 | 0.0046 |
| **NK cells density (stroma)** | 0.0078 | 0.0043 | 0.0108 | 0.0571 | 0.0013 | 0.0584 | 0.0029 | 0.0043 | 0.0074 |
| **Treg density (tumour)** | 0.0054 | 0.0025 | 0.0078 | 0.0344 | 0.0004 | 0.0349 | 0.0014 | 0.0025 | 0.0054 |
| **Tregs density (stroma)** | 0.0093 | 0.0048 | 0.0124 | 0.0559 | 0.0014 | 0.0573 | 0.0030 | 0.0048 | 0.0092 |
| **Th density (tumour)** | 0.0076 | 0.0022 | 0.0107 | 0.0406 | 0.0001 | 0.0407 | 0.0016 | 0.0022 | 0.0070 |
| **Th density (stroma)** | 0.0131 | 0.0050 | 0.0190 | 0.0776 | 0.0004 | 0.0781 | 0.0028 | 0.0050 | 0.0097 |
| **B cells density (tumour)** | 0.0107 | 0.0043 | 0.0170 | 0.0845 | 0.0011 | 0.0856 | 0.0030 | 0.0043 | 0.0105 |
| **B cells density (stroma)** | 0.0154 | 0.0068 | 0.0260 | 0.1327 | 0.0018 | 0.1345 | 0.0034 | 0.0068 | 0.0153 |
| **cCASP3+Tc (tumour) %** | 37.5113 | 42.1650 | 19.2926 | 62.6800 | 1.4800 | 64.1600 | 22.1100 | 42.1650 | 53.8700 |
| **cCASP3+ Tc (stroma) %** | 55.3719 | 66.3450 | 23.8371 | 79.8900 | 5.6300 | 85.5200 | 35.8150 | 66.3450 | 73.8250 |
| **cCASP8+Tc (tumour) %** | 35.4457 | 35.7454 | 18.2542 | 58.0400 | 10.0700 | 68.1100 | 17.9239 | 35.7454 | 50.0844 |
| **cCASP8+ Tc (stroma) %** | 55.4231 | 62.2900 | 23.1225 | 74.4800 | 12.8100 | 87.2900 | 37.0550 | 62.2900 | 75.6600 |
| **cCASP3+ NK (tumour) %** | 37.8122 | 38.5950 | 11.8429 | 48.5800 | 14.5500 | 63.1300 | 27.2175 | 38.5950 | 47.3075 |
| **cCASP3+ NK (stroma) %** | 57.8128 | 59.4800 | 11.8980 | 48.4600 | 33.1200 | 81.5800 | 49.3925 | 59.4800 | 65.9050 |
| **cCASP3+ Tregs (tumour) %** | 48.0819 | 47.2700 | 10.7320 | 50.3300 | 17.0600 | 67.3900 | 43.4525 | 47.2700 | 53.5250 |
| **cCASP3+ Tregs (stroma) %** | 69.6025 | 71.3600 | 10.1422 | 48.9000 | 36.1500 | 85.0500 | 66.2600 | 71.3600 | 76.0225 |
| **cCASP3+ Th (tumour) %** | 48.8797 | 49.4050 | 14.6297 | 74.9900 | 13.3500 | 88.3400 | 40.0575 | 49.4050 | 56.2400 |
| **cCASP3+ Th (stroma) %** | 67.5247 | 69.0900 | 12.1551 | 60.0900 | 23.3000 | 83.3900 | 62.7775 | 69.0900 | 74.6950 |
| **cCASP3+ B cells (tumour) %** | 50.9672 | 52.5500 | 10.7839 | 44.1900 | 26.9200 | 71.1100 | 44.1825 | 52.5500 | 57.9900 |
| **cCASP3+ B cells (stroma) %** | 74.8975 | 75.9950 | 8.8994 | 30.1400 | 57.7700 | 87.9100 | 66.8675 | 75.9950 | 82.2325 |
| **cCASP3-Tc (tumour) density** | 0.0037 | 0.0016 | 0.0062 | 0.0314 | 0.0002 | 0.0316 | 0.0008 | 0.0016 | 0.0036 |
| **cCASP3- Tc (stroma) density** | 0.0043 | 0.0017 | 0.0065 | 0.0293 | 0.0004 | 0.0297 | 0.0011 | 0.0017 | 0.0047 |
| **cCASP8-Tc (tumour) density** | 0.0032 | 0.0018 | 0.0037 | 0.0200 | 0.0000 | 0.0200 | 0.0008 | 0.0018 | 0.0037 |
| **cCASP8- Tc (stroma) density** | 0.0034 | 0.0022 | 0.0036 | 0.0200 | 0.0000 | 0.0200 | 0.0010 | 0.0022 | 0.0047 |
| **cCASP3- NK (tumour) density** | 0.0024 | 0.0016 | 0.0026 | 0.0136 | 0.0005 | 0.0142 | 0.0010 | 0.0016 | 0.0031 |
| **cCASP3- NK (stroma) density** | 0.0029 | 0.0017 | 0.0036 | 0.0200 | 0.0006 | 0.0207 | 0.0012 | 0.0017 | 0.0033 |
| **cCASP3- Tregs (tumour) density** | 0.0025 | 0.0014 | 0.0036 | 0.0183 | 0.0003 | 0.0186 | 0.0007 | 0.0014 | 0.0026 |
| **cCASP3- Tregs (stroma) density** | 0.0023 | 0.0014 | 0.0025 | 0.0128 | 0.0005 | 0.0133 | 0.0010 | 0.0014 | 0.0024 |
| **cCASP3- Th (tumour) density** | 0.0035 | 0.0015 | 0.0050 | 0.0223 | 0.0001 | 0.0224 | 0.0009 | 0.0015 | 0.0033 |
| **cCASP3- Th (stroma) density** | 0.0034 | 0.0018 | 0.0040 | 0.0132 | 0.0002 | 0.0134 | 0.0009 | 0.0018 | 0.0033 |
| **cCASP3- B cells (tumour) density** | 0.0044 | 0.0024 | 0.0064 | 0.0336 | 0.0007 | 0.0343 | 0.0013 | 0.0024 | 0.0048 |
| **cCASP3- B cells (stroma) density** | 0.0028 | 0.0017 | 0.0032 | 0.0168 | 0.0004 | 0.0173 | 0.0010 | 0.0017 | 0.0036 |

**Table S5. Multiple comparisons between cCASP3+ lymphocytes in OSCC (tumour and stroma) and control [intraepithelial (IE) and stroma].**

| **1 OSCC: Intratumoural (T) cCASP3+ Lymphocytes comparisons** | | | | |
| --- | --- | --- | --- | --- |
| **Šidák's multiple comparisons test** | **Mean Diff.** | **95% Confidence Interval** | **p-value summary** | **p-value** |
| Tc (T) versus Tregs (T) | −10.5700 | −20.40 to −0.74 | ***** | **0.0262** |
| Tc (T) versus Th (T) | −11.3700 | −21.20 to −1.54 | ***** | **0.0125** |
| Tc (T) versus B cells (T) | −13.4600 | −23.28 to −3.62 | ****** | **0.0015** |
| Tc (T) versus NK cells (T) | −0.3009 | −10.13 to 9.52 | ns | >0.9999 |
| Tregs (T) versus Th (T) | −0.7978 | −10.63 to 9.03 | ns | >0.9999 |
| Tregs (T) versus B cells (T) | −2.8850 | −12.71 to 6.94 | ns | 0.9945 |
| Tregs (T) versus NK cells (T) | 10.2700 | 0.44 to 20.10 | ***** | **0.0342** |
| Th (T) versus NK cells (T) | 11.0700 | 1.24 to 20.90 | ***** | **0.0166** |
| B cells (T) versus NK cells (T) | 13.1600 | 3.32 to 22.98 | ****** | **0.0021** |
| Th (T) versus B cells (T) | −2.0880 | −11.92 to 7.74 | ns | 0.9996 |

| **2 OSCC: Stromal (S) cCASP3+ Lymphocytes comparisons** | | | | |
| --- | --- | --- | --- | --- |
| **Šidák's multiple comparisons test** | **Mean Diff.** | **95% Confidence Interval** | **p-value summary** | **p-value** |
| Tc (S) versus Tregs (S) | −14.2300 | −24.47 to −3.99 | ****** | **0.0012** |
| Tc (S) versus Th (S) | −12.1500 | −22.39 to −1.91 | ****** | **0.0094** |
| Tc (S) versus B (S) | −19.5300 | −29.76 to −9.28 | ******** | **<0.0001** |
| Tc (S) versus NK (S) | −2.4410 | −12.68 to 7.79 | ns | 0.9990 |
| Tregs (S) versus Th (S) | 2.0780 | −8.16 to 12.32 | ns | 0.9998 |
| Tregs (S) versus B cells (S) | −5.2950 | −15.53 to 4.94 | ns | 0.7885 |
| Tregs (S) versus NK cells (S) | 11.7900 | 1.55 to 22.03 | ***** | **0.0132** |
| Th (S) versus B cells (S) | −7.3730 | −17.61 to 2.86 | ns | 0.3524 |
| Th (S) versus NK cells (S) | 9.7120 | −0.52 to 19.95 | ns | 0.0756 |
| B cells (S) versus NK cells (S) | 17.0800 | 6.84 to 27.32 | ******** | **<0.0001** |

| **3 OSCC: Intratumour (T) versus stroma (S) cCASP3+ Lymphocytes comparisons** | | | | |
| --- | --- | --- | --- | --- |
| **Šidák's multiple comparisons test** | **Mean Diff.** | **95% Confidence Interval** | **p-value summary** | **p-value** |
| Tc (T) versus Tc (S) | −17.8600 | −26.99 to −8.72 | ******** | **<0.0001** |
| Tregs (T) versus Tregs (S) | −21.5200 | −30.65 to −12.39 | ******** | **<0.0001** |
| Th (T) versus Th (S) | −18.6500 | −27.78 to −9.51 | ******** | **<0.0001** |
| B cells (T) versus B cells (S) | −23.9300 | −33.06 to −14.80 | ******** | **<0.0001** |
| NK cells (T) versus NK cells (S) | −20.0000 | −29.13 to −10.87 | ******** | **<0.0001** |

| **4 Control: Intraepithelial (IE) cCASP3+ Lymphocytes comparisons** | | | | |
| --- | --- | --- | --- | --- |
| **Šidák's multiple comparisons test** | **Mean Diff.** | **95% Confidence Interval** | **p-value summary** | **p-value** |
| Tc (IE) versus Tregs (IE) | −5.7840 | −25.11 to 13.54 | ns | 0.9997 |
| Tc (IE) versus Th (IE) | −8.0220 | −26.24 to 10.20 | ns | 0.9680 |
| Tc (IE) versus B (IE) | −0.9540 | −19.18 to 17.27 | ns | >0.9999 |
| Tc (IE) versus NK (IE) | −6.1800 | −24.40 to 12.04 | ns | 0.9982 |
| Tregs (IE) versus Th (IE) | −2.2390 | −21.57 to 17.09 | ns | >0.9999 |
| Tregs (IE) versus B (IE) | 4.8300 | −14.50 to 24.16 | ns | >0.9999 |
| Tregs (IE) versus NK (IE) | −0.3965 | −19.72 to 18.93 | ns | >0.9999 |
| Th (IE) versus B (IE) | 7.0680 | −11.15 to 25.29 | ns | 0.9912 |
| Th (IE) versus NK (IE) | 1.8420 | −16.38 to 20.06 | ns | >0.9999 |
| B (IE) versus NK (IE) | −5.2260 | −23.45 to 13.00 | ns | 0.9998 |

| **5 Control: Stroma (S) cCASP3+ Lymphocytes comparisons** | | | | |
| --- | --- | --- | --- | --- |
| **Šidák's multiple comparisons test** | **Mean Diff.** | **95% Confidence Interval** | **p-value summary** | **p-value** |
| Tc (S) versus Tregs (S) | −12.5700 | −30.46 to 5.32 | ns | 0.3594 |
| Tc (S) versus Th (S) | −15.5300 | −32.40 to 1.33 | ns | 0.0901 |
| Tc (S) versus B (S) | −13.3100 | −30.18 to 3.55 | ns | 0.2183 |
| Tc (S) versus NK (S) | −7.9440 | −24.81 to 8.92 | ns | 0.8443 |
| Tregs (S) versus Th (S) | −2.9610 | −20.85 to 14.93 | ns | >0.9999 |
| Tregs (S) versus B (S) | −0.7425 | −18.63 to 17.15 | ns | >0.9999 |
| Tregs (S) versus NK (S) | 4.6250 | −13.26 to 22.51 | ns | 0.9973 |
| Th (S) versus B (S) | 2.2180 | −14.65 to 19.08 | ns | >0.9999 |
| Th (S) versus NK (S) | 7.5860 | −9.28 to 24.45 | ns | 0.8774 |
| B (S) versus NK (S) | 5.3680 | −11.50 to 22.23 | ns | 0.9866 |

| **6 Control: Intraepithelial (IE) versus stroma (S) cCASP3+ Lymphocytes comparisons** | | | | |
| --- | --- | --- | --- | --- |
| **Šidák's multiple comparisons test** | **Mean Diff.** | **95% Confidence Interval** | **p-value summary** | **p-value** |
| Tc (IE) versus Tc (S) | −21.7400 | −39.97 to −3.52 | ****** | **0.0088** |
| Tregs (IE) versus Tregs (S) | −28.5300 | −48.90 to −8.15 | ****** | **0.0012** |
| Th (IE) versus Th (S) | −29.2500 | −47.47 to −11.03 | ******* | **0.0002** |
| B (IE) versus B (S) | −34.1000 | −52.32 to −15.88 | ******** | **<0.0001** |
| NK (IE) versus NK (S) | −23.5100 | −41.73 to −5.28 | ****** | **0.0035** |

**Table S6. Caspase-8 activation in CASP8 (mutated) or (wt) cells upon FasL (400 ng/ml) treatment and Caspase-3/7 activation in CASP8 (mutated) or (wt) cells upon FasL (400 ng/ml) treatment.**

**Caspase-8 activation in CASP8 (mutated) or (wt) cells upon FasL (400 ng/ml) treatment**

| **Tukey's multiple comparisons test** | **Mean Diff.** | **95% Confidence Interval** | **p-value summary** | **p-value** |
| --- | --- | --- | --- | --- |
| Oral keratinocytes versus SJG17 (WT) | −0.9565 | −2.48 to 0.57 | ns | 0.4394 |
| Oral keratinocytes versus SJG33 (WT) | 1.8830 | 0.27 to 3.49 | ***** | **0.0136** |
| Oral keratinocytes versus SJG6 (missense) | 1.8830 | 0.27 to 3.49 | ***** | **0.0136** |
| Oral keratinocytes versus SJG18 (missense) | 2.2540 | 0.68 to 3.82 | ****** | **0.0013** |
| Oral keratinocytes versus SJG13 (nonsense) | 2.3180 | 0.75 to 3.88 | ******* | **0.0009** |
| SJG17 (WT) versus SJG33 (wt) | 2.8390 | 1.31 to 4.36 | ******** | **<0.0001** |
| SJG17 (WT) versus SJG6 (missense) | 2.8390 | 1.31 to 4.36 | ******** | **<0.0001** |
| SJG17 (WT) versus SJG18 (missense) | 3.2100 | 1.72 to 4.69 | ******** | **<0.0001** |
| SJG17 (WT) versus SJG13 (nonsense) | 3.2750 | 1.79 to 4.75 | ******** | **<0.0001** |
| SJG33 (WT) versus SJG6 (missense) | 0.0000 | −1.61 to 1.61 | ns | >0.9999 |
| SJG33 (WT) versus SJG18 (missense) | 0.3708 | −1.19 to 1.93 | ns | 0.9806 |
| SJG33 (WT) versus SJG13 (nonsense) | 0.4354 | −1.13 to 2.00 | ns | 0.9611 |
| SJG6 (missense) versus SJG18 (missense) | 0.3708 | −1.19 to 1.93 | ns | 0.9806 |
| SJG6 (missense) versus SJG13 (nonsense) | 0.4354 | −1.13 to 2.00 | ns | 0.9611 |
| SJG18 (missense) versus SJG13 (nonsense) | 0.0646 | −1.45 to 1.58 | ns | >0.9999 |

ns: not significant. Values in bold are statistically significant.

**Caspase-3/7 activation in CASP8 (mutated) or (WT) cells upon FasL (400 ng/ml) treatment**

| **Tukey's multiple comparisons test** | **Mean Diff.** | **95% Confidence Interval** | **p-value summary** | **p-value** |
| --- | --- | --- | --- | --- |
| Oral keratinocytes versus SJG17 (WT) | 0.9496 | −0.01 to 1.91 | ns | 0.0568 |
| Oral keratinocytes versus SJG33 (WT) | 1.0340 | 0.08 to 1.98 | ***** | **0.0248** |
| Oral keratinocytes versus SJG6 (missense) | 1.4650 | 0.39 to 2.53 | ****** | **0.0024** |
| Oral keratinocytes versus SJG18 (missense) | 1.4500 | 0.38 to 2.51 | ****** | **0.0027** |
| Oral keratinocytes versus SJG13 (nonsense) | 1.5830 | 0.65 to 2.51 | ******* | **0.0001** |
| SJG17 (WT) versus SJG33 (WT) | 0.0846 | −0.79 to 0.96 | ns | 0.9997 |
| SJG17 (WT) versus SJG6 (missense) | 0.5150 | −0.49 to 1.52 | ns | 0.6544 |
| SJG17 (WT) versus SJG18 (missense) | 0.5000 | −0.51 to 1.51 | ns | 0.6820 |
| SJG17 (WT) versus SJG13 (nonsense) | 0.6330 | −0.22 to 1.49 | ns | 0.2640 |
| SJG33 (WT) versus SJG6 (missense) | 0.4304 | −0.56 to 1.42 | ns | 0.7858 |
| SJG33 (WT) versus SJG18 (missense) | 0.4154 | −0.57 to 1.40 | ns | 0.8096 |
| SJG33 (WT) versus SJG13 (nonsense) | 0.5484 | −0.28 to 1.38 | ns | 0.3867 |
| SJG6 (missense) versus SJG18 (missense) | −0.0151 | −1.12 to 1.09 | ns | >0.9999 |
| SJG6 (missense) versus SJG13 (nonsense) | 0.1179 | −0.85 to 1.09 | ns | 0.9991 |
| SJG18 (missense) versus SJG13 (nonsense) | 0.1330 | −0.84 to 1.10 | ns | 0.9985 |
|  |  |  |  |  |

ns: not significant. Values in bold are statistically significant.

**Table S7. COX regression analysis between tumour and TILs parameters with Disease Specific Survival (DSS).**

| **Tumour parameter** | ***P* value summary** | ***P* value** | **Exp (B)** | **95% Confidence Interval** | |
| --- | --- | --- | --- | --- | --- |
|  |  |  |  | **Lower bound** | **Upper bound** |
| **cCASP3+ tumour** | ***** | **0.0182** | 3.619 | 1.245 | 10.520 |
| **cCASP8+ tumour** | ns | 0.1800 | 1.939 | 0.726 | 5.177 |
| **PDL1+ tumour** | ns | 0.4300 | 1.476 | 0.561 | 3.886 |
| **FasL+ tumour** | ns | 0.0700 | 1.020 | 0.998 | 1.043 |
| **FasL+ Stroma** | ns | 0.1000 | 1.014 | 0.997 | 1.031 |
| **Node metastasis** | ns | 0.2400 | 1.825 | 0.659 | 5.051 |

| **Lymphocyte Parameter** | ***P* value summary** | ***P* value** | **Exp (B)** | **95% Confidence Interval** | |
| --- | --- | --- | --- | --- | --- |
|  |  |  |  | **Lower bound** | **Upper bound** |
| **cCASP3+Tc (tumour)** | ns | 0.6830 | 1.005 | 0.981 | 1.029 |
| **cCASP8+Tc (tumour)** | ns | 0.3370 | 1.016 | 0.983 | 1.050 |
| **cCASP3+ Tc (stroma)** | ns | 0.6740 | 1.004 | 0.985 | 1.024 |
| **cCASP8+ Tc (stroma)** | ns | 0.3280 | 1.013 | 0.987 | 1.040 |
| **cCASP3+ NK (tumour)** | ns | 0.7490 | 0.993 | 0.949 | 1.039 |
| **cCASP3+ NK (stroma)** | ns | 0.8330 | 1.004 | 0.964 | 1.047 |
| **cCASP3+ Tregs (tumour)** | ns | 0.8130 | 1.007 | 0.947 | 1.071 |
| **cCASP3+ Tregs (stroma)** | ns | 0.7830 | 0.992 | 0.939 | 1.048 |
| **cCASP3+ Th (tumour)** | ns | 0.5970 | 0.990 | 0.956 | 1.026 |
| **cCASP3+ Th (stroma)** | ns | 0.5580 | 1.018 | 0.958 | 1.082 |
| **cCASP3+ B cells (tumour)** | ns | 0.9220 | 1.002 | 0.964 | 1.042 |
| **cCASP3+ B cells (stroma)** | ns | 0.4450 | 0.979 | 0.927 | 1.034 |
| **Tc density (tumour)** | ns | 0.8650 | 0.005 | 0.000 | 7E+23 |
| **Tc density (stroma)** | ns | 0.9550 | 2.825 | 0.000 | 2E+16 |
| **NK cells density (tumour)** | ns | 0.5700 | 0.000 | 0.000 | 4E+26 |
| **NK cells density (stroma)** | ns | 0.0930 | 2E+77 | 0.000 | 3E+167 |
| **Treg density (tumour)** | ns | 0.8280 | 1E+12 | 0.000 | 1E+122 |
| **Tregs density (stroma)** | ns | 0.7520 | 1E+11 | 0.000 | 1E+80 |
| **Th density (tumour)** | ns | 0.5920 | 0E+00 | 0.000 | 3E+45 |
| **Th density (stroma)** | ns | 0.3820 | 1E+19 | 0.000 | 6E+61 |
| **B cells density (tumour)** | ns | 0.2540 | 0E+00 | 0.000 | 2E+28 |
| **B cells density (stroma)** | ns | 0.1770 | 9E+30 | 0.000 | 7E+75 |

ns: not significant. Values in bold are statistically significant. Exp (B): Hazard ratio/ associated risk

**Table S8. Multinominal logistic regression analysis**

| **A** |  | ***P* value summary** | ***P* value** | **Exp(B)** | **95% Confidence Interval** | |
| --- | --- | --- | --- | --- | --- | --- |
| **Compare parameter** |  |  |  |  | **Lower bound** | **Upper bound** |
| **Node positive** | **cCASP3+Tc (tumour)** | ***** | 0.0390 | 1.0460 | 1.0020 | 1.0910 |
|  | **cCASP3+ Tc (stroma)** | ns | 0.0750 | 1.0310 | 0.9970 | 1.0650 |
|  | **cCASP3+ NK (tumour)** | ns | 0.3680 | 1.0290 | 0.9670 | 1.0940 |
|  | **cCASP3+ NK (stroma)** | ns | 0.7050 | 0.9880 | 0.9310 | 1.0500 |
|  | **cCASP3+ Tregs (tumour)** | ns | 0.2760 | 1.0400 | 0.9690 | 1.1170 |
|  | **cCASP3+ Tregs (stroma)** | ns | 0.5270 | 1.0230 | 0.9530 | 1.0990 |
|  | **cCASP3+ Th (tumour)** | ns | 0.5750 | 0.9860 | 0.9380 | 1.0360 |
|  | **cCASP3+ Th (stroma)** | ns | 0.5610 | 0.9820 | 0.9230 | 1.0450 |
|  | **cCASP3+ B cells (tumour)** | ns | 0.5620 | 1.0200 | 0.9540 | 1.0900 |
|  | **cCASP3+ B cells (stroma)** | ns | 0.3750 | 0.9630 | 0.8870 | 1.0460 |
|  |  |  |  |  |  |  |
| **B** |  | ***P* value summary** | ***P* value** | **Exp(B)** | **95% Confidence Interval** | |
| **Compare parameter** |  |  |  |  | **Lower bound** | **Upper bound** |
| **Node positive** | **Tc density (tumour)** | ns | 0.3670 | 6.01E+15 | 0.0000 | 1.21E+50 |
|  | **Tc density (stroma)** | ns | 0.9020 | 9.3300 | 0.0000 | 2.39E+16 |
|  | **NK cells density (tumour)** | ns | 0.3270 | 0.3490 | 0.0420 | 2.8670 |
|  | **NK cells density (stroma)** | ns | 0.3650 | 0.0000 | 0.0000 | 6.71E+18 |
|  | **Treg density (tumour)** | ns | 0.3490 | 0.0000 | 0.0000 | 3.51E+22 |
|  | **Tregs density (stroma)** | ns | 0.2650 | 0.0000 | 0.0000 | 6.28E+12 |
|  | **Th density (tumour)** | ns | 0.7010 | 7.10E+05 | 0.0000 | 4.81E+35 |
|  | **Th density (stroma)** | ns | 0.5840 | 0.0000 | 0.0000 | 5.46E+11 |
|  | **B cells density (tumour)** | ns | 0.4370 | 0.0000 | 0.0000 | 8.28E+11 |
|  | **B cells density (stroma)** | ns | 0.2910 | 0.0000 | 0.0000 | 3.24E+07 |
|  |  |  |  |  |  |  |
| **C** |  | ***P* value summary** | ***P* value** | **Exp(B)** | **95% Confidence Interval** | |
| **Compare parameter** |  |  |  |  | **Lower bound** | **Upper bound** |
| **Tumour FasL expression** | **cCASP3+Tc (tumour)** | ns | 0.5570 | 0.9870 | 0.9440 | 1.0310 |
|  | **cCASP3+ Tc (stroma)** | ns | 0.6620 | 0.9920 | 0.9590 | 1.0270 |
|  | **cCASP3+ NK (tumour)** | ns | 0.7630 | 1.0120 | 0.9390 | 1.0900 |
|  | **cCASP3+ NK (stroma)** | ns | 0.7830 | 1.0110 | 0.9340 | 1.0950 |
|  | **cCASP3+ Tregs (tumour)** | ns | 0.9680 | 1.0020 | 0.9220 | 1.0890 |
|  | **cCASP3+ Tregs (stroma)** | ns | 0.9850 | 1.0010 | 0.9180 | 1.0910 |
|  | **cCASP3+ Th (tumour)** | ns | 0.4230 | 0.9760 | 0.9200 | 1.0360 |
|  | **cCASP3+ Th (stroma)** | ns | 0.2160 | 0.9580 | 0.8950 | 1.0250 |
|  | **cCASP3+ B cells (tumour)** | ns | 0.2670 | 1.0550 | 0.9600 | 1.1580 |
|  | **cCASP3+ B cells (stroma)** | ns | 0.2460 | 1.0690 | 0.9550 | 1.1980 |

ns: not significant, Exp (B): odds ratio/ associated risk

**Table S9: Multiple comparisons between cCASP3+Tc in OSCC (tumour and stroma), Dysplasia (Intraepithelial (lE) and stroma) and Control (IE and stroma).**

| **Šidák’s multiple comparisons test** | **Mean Diff.** | **95% Confidence Interval** | ***P* value summary** | **p-value** |
| --- | --- | --- | --- | --- |
| OSCC (IE) versus OSCC (S) | −17.8600 | −30.90 to −4.81 | ****** | **0.0018** |
| Dysplasia (IE) versus dysplasia (S) | −28.5300 | −49.00 to −8.06 | ****** | **0.0014** |
| Control (IE) versus control (S) | −21.7400 | −54.74 to 11.25 | ns | 0.4553 |
| OSCC (IE) versus dysplasia (IE) | −6.0260 | −23.19 to 11.13 | ns | 0.9700 |
| OSCC (IE) versus control (IE) | 0.5972 | −24.49 to 25.69 | ns | >0.999 |
| Dysplasia (IE) versus control (IE) | 6.6240 | −20.83 to 34.08 | ns | 0.9979 |
| OSCC (S) versus dysplasia (S) | −16.7000 | −33.86 to 0.46 | ns | 0.0618 |
| OSCC (S) versus control (S) | −3.2860 | −28.38 to 21.80 | ns | >0.999 |
| Dysplasia (S) versus control (S) | 13.4100 | −14.04 to 40.87 | ns | 0.8131 |

ns: not significant, IE: intraepithelial, S: stroma. Values in bold are statistically significant.
